# Brain re-expansion rate on the first postoperative day and risk of symptomatic recurrence after burr-hole drainage for chronic subdural hematoma

**DOI:** 10.3389/fsurg.2026.1949842

**Published:** 2026-10-21

**Authors:** Yinxing Sheng, Jinfeng Pang, Wen Wang

**Affiliations:** 1 Department of Anesthesiology, Affiliated Hospital of Beihua University, Jilin City, Jilin Province, China; 2 Department of Neurology, Affiliated Hospital of Beihua University, Jilin City, Jilin Province, China; 3 Department of Anesthesiology, Huai’an Hospital of Huai’an City (Huai’an Clinical Medical College of Jiangsu University), Huai’an City, Jiangsu Province, China

**Keywords:** brain re-expansion, chronic subdural hematoma, competing risks, computed tomography, recurrence

## Abstract

**Background:**

Early brain re-expansion may indicate recurrence risk after chronic subdural hematoma evacuation. This study examined its association with 90-day symptomatic recurrence and its incremental prognostic value.

**Methods:**

This retrospective study included 286 adults undergoing first-time burr-hole evacuation with closed drainage. Volumes on preoperative computed tomography and postoperative imaging at 22–26 h were used to standardize re-expansion to 24 h. Symptomatic recurrence required clinical deterioration, radiographic progression, and reintervention. Follow-up began at postoperative imaging and ended by postoperative day 90. Cause-specific Cox and Fine–Gray models used joint strata for brain atrophy and antithrombotic use, with additional adjustment for age, bilateral disease, and preoperative volume. Internal validation used 1,000 patient-level bootstrap samples with the stratum structure held fixed.

**Results:**

Symptomatic recurrence occurred in 47 patients (16.4%); eight died before recurrence and 231 remained alive without recurrence. Each 10-percentage-point/24 h increase in re-expansion was associated with a lower cause-specific hazard (adjusted hazard ratio, 0.74; 95% confidence interval, 0.64–0.85) and subdistribution hazard (adjusted subdistribution hazard ratio, 0.74; 95% confidence interval, 0.65–0.85; both *P* < 0.001). Adding re-expansion increased the within-stratum Harrell concordance index from 0.657 to 0.695 (difference, 0.039; 95% confidence interval, −0.015 to 0.092; *P* = 0.155). The 90-day area under the receiver operating characteristic curve increased from 0.731 to 0.774 (difference, 0.042; 95% confidence interval, 0.001–0.083; *P* = 0.043). The full Fine–Gray model had an optimism-corrected Brier score of 0.116 and calibration slope of 0.89.

**Conclusions:**

Faster first-day brain re-expansion was associated with lower symptomatic recurrence hazard after accounting for competing death. The internally observed increase in 90-day discrimination requires external validation before clinical application.

## 1 Introduction

Chronic subdural hematoma (CSDH) predominantly affects older adults, and its diagnosis and treatment impose a substantial healthcare burden on this population (1). Clinical manifestations include headache, gait disturbance, cognitive decline, focal neurological deficits, and impaired consciousness; comorbidities and differences in baseline function complicate diagnosis, treatment, and prognostic assessment (2). Inflammation, angiogenesis, and fibrinolysis following injury to the dural border contribute to hematoma persistence, and leakage from microvessels in the outer membrane may promote recurrent accumulation of subdural contents (3).

Burr-hole irrigation with closed drainage is a commonly used treatment for patients with symptoms or substantial mass effect, although management details vary, including the number of burr holes, drain location, and drainage duration (4). Residual fluid, air, and brain re-expansion after hematoma evacuation jointly determine the early postoperative configuration of the subdural space; decompression and cavity closure follow different time courses (5). Clinical evidence relating irrigation fluid temperature to postoperative recurrence suggests that perioperative management may also influence subsequent hematoma evolution (6).

Recurrence requiring further treatment may still occur after burr-hole drainage, and differences in outcome definitions and follow-up duration affect comparisons across studies (7). Computed tomography (CT) and magnetic resonance imaging can depict heterogeneity in hematoma contents, membranous structures, and residual cavities (8). The association between internal hematoma architecture and recurrence suggests that preoperative lesion characteristics may influence evolution after evacuation (9). Frailty is associated with mortality and other adverse outcomes in patients with CSDH, making competing death an important consideration when evaluating recurrence (10). Relative volume change during the first postoperative day is also affected by the interval to imaging; a clearly defined, time-standardized measurement is therefore essential for evaluating this parameter.

This study used completion of the first postoperative CT scan as the landmark to evaluate the association between the standardized 24 h brain re-expansion rate and symptomatic recurrence through postoperative day 90. The primary analysis estimated the cause-specific instantaneous hazard among patients who were alive and recurrence-free. Cumulative incidence and subdistribution hazard associations in the presence of competing death were also evaluated, together with the incremental prognostic information provided by this imaging parameter beyond prespecified clinical and imaging variables.

## 2 Materials and methods

### 2.1 Study design and participants

This retrospective study consecutively included adults with CSDH who underwent first-time burr-hole irrigation with closed drainage at our hospital between October 1, 2021, and September 30, 2025. Outcome ascertainment was completed by December 29, 2025. Reporting followed the relevant items of the Strengthening the Reporting of Observational Studies in Epidemiology (STROBE) statement (11) (Supplementary Table S1).

Neurological status at admission was characterized using the Markwalder grade and Glasgow Coma Scale (GCS), which recorded symptoms and level of consciousness, respectively (12). CSDH was diagnosed on the basis of clinical manifestations and subdural collections on cranial CT. In patients with atypical imaging findings, the signal characteristics of the contents, membranous structures, and enhancement patterns on magnetic resonance imaging were considered to distinguish other subdural lesions (13).

The inclusion criteria were as follows: (1) age ≥18 years; (2) unilateral or bilateral CSDH confirmed on preoperative cranial CT; (3) cranial CT suitable for volumetric analysis obtained within 24 h before surgery; (4) the first postoperative cranial CT obtained 22–26 h after surgery; (5) preoperative and postoperative CT slice thickness ≤5 mm, with complete original Digital Imaging and Communications in Medicine (DICOM) datasets; (6) complete drainage records for the first 0–24 h after surgery; and (7) no repeat surgery for the hematoma before the landmark defined by the first postoperative CT.

The exclusion criteria were as follows: (1) acute or subacute subdural hematoma, or isolated subdural hygroma; (2) previous CSDH surgery, or use of craniotomy, endoscopic surgery, twist-drill drainage, or another procedure for the index operation; (3) concomitant acute traumatic brain injury, intracranial tumor, recent large cerebral infarction, or another lesion potentially affecting intracranial volumetric assessment; (4) acute intracranial hemorrhage or repeat surgery before the first postoperative CT; and (5) inadequate quality of key images, missing primary-model variables, or inability to ascertain the 90-day postoperative outcome. The sample size was determined by the number of eligible patients during the study period. Only one reason for exclusion was recorded for each patient according to a prespecified hierarchical order, yielding a final sample of 286 patients.

The study protocol was approved by the Medical Ethics Committee of the Affiliated Hospital of Beihua University on July 1, 2024 (approval number: 20240060). Patient identification, data extraction, imaging measurements, statistical analyses, and telephone outcome verification were conducted after approval. Data were de-identified before analysis, and the ethics committee waived written informed consent because the retrospective medical-record review involved minimal risk. Telephone verification was performed by authorized personnel, and only information required for outcome adjudication was recorded. Coded identifiers and restricted access were used for the study database. The study was conducted in accordance with the Declaration of Helsinki.

### 2.2 Surgery and perioperative management

All patients underwent irrigation through one or two burr holes, with placement of a closed subdural drain on the operated side. Patients with bilateral disease underwent unilateral or simultaneous bilateral surgery according to their clinical symptoms, hematoma volume, and mass effect. Drains were routinely maintained for at least 24 h; removal was determined by the characteristics and volume of the drainage fluid, neurological status, and follow-up imaging. Drainage volume during the first 0–24 h after surgery was defined as the cumulative volume documented in nursing records from the end of surgery to 24 h postoperatively. Recorded variables included anesthetic technique, number of burr holes, operated side, drainage duration, drainage volume during the first 0–24 h, duration of preoperative antiplatelet or anticoagulant interruption, and timing of postoperative medication resumption.

Postoperative mobilization followed the ward's graded mobilization protocol. During the first 0–6 h, activity consisted mainly of repositioning in bed and limb exercises. For patients with stable circulatory, respiratory, and neurological status and a patent drainage system, the head of the bed was gradually elevated and sitting was initiated after 6 h, followed by assisted bedside standing or short-distance walking within 12–24 h. The closed drainage system was maintained during transfer and out-of-bed activity, with the drainage bag positioned 10–15 cm below the level of the external auditory canal. When temporary drain clamping was required for safety, each episode lasted ≤10 min, and gravity drainage was resumed immediately upon the patient's return to bed. Out-of-bed mobilization was delayed in patients with a reduced level of consciousness, new focal neurological deficits, symptomatic hypotension, active bleeding, or a high risk of falls.

### 2.3 Clinical and imaging data

Two trained investigators independently extracted data using a standardized form. Variables included age, sex, body mass index, smoking history, alcohol consumption, recent head trauma, hypertension, diabetes mellitus, coronary artery disease, previous stroke, preoperative antithrombotic use, admission Markwalder grade, GCS score, platelet count, international normalized ratio, and activated partial thromboplastin time.

Preoperative CT variables included hematoma laterality, hematoma volume on the operated side, maximum thickness, midline shift, density, and internal architecture. Density was classified as hypodense, isodense, hyperdense, or mixed, and internal architecture as homogeneous, laminar, separated, or trabecular. Thickness, midline shift, and residual subdural components were assessed with reference to published imaging methods, using a consistent measurement protocol within this study (14). Brain atrophy was assessed using the global cortical atrophy (GCA) visual grading scale, with grades 0, 1, 2, and 3 representing no evident atrophy, mild atrophy, moderate atrophy, and severe atrophy, respectively. GCA ≥2 was classified as moderate-to-severe brain atrophy (15). For simultaneous bilateral surgery, preoperative hematoma volume was the sum of both sides; for bilateral disease treated unilaterally, only the operated side was measured.

### 2.4 Twenty-four-hour postoperative brain re-expansion rate

DICOM data from the preoperative and first postoperative CT scans were imported into 3D Slicer, and three-dimensional volumes were obtained by semiautomatic segmentation with slice-by-slice manual correction. CT segmentation can quantify the extent of irregular subdural collections (16). A uniform brain window was used, with a window width of 80 Hounsfield units (HU) and a window level of 40 HU; mixed-density components were reassessed using a width of 200 HU and a level of 50 HU. Initial thresholds were −1,000 to −200 HU for air and −20 to 100 HU for residual fluid or hematoma. Partial-volume regions between −200 and −20 HU, boundaries adjacent to the inner table of the skull or venous sinuses, and slices affected by artifacts were manually corrected using axial, coronal, sagittal, and adjacent-slice views. Final boundaries were determined by anatomical continuity and multiplanar consistency. Areas of disagreement were jointly reviewed by the two assessors.

Preoperative hematoma volume on the operated side (*V*_pre_, mL) and total postoperative residual subdural volume (*V*_post_, mL) were measured. The latter was the sum of residual fluid or hematoma and subdural air volumes. Volumes were summed across both sides for simultaneous bilateral surgery, whereas only the operated side was included for bilateral disease treated unilaterally. The basic expression for relative volume change was (*V*_pre_ − *V*_post_)/*V*_pre_ × 100% (17).

The standardized 24 h brain re-expansion rate was defined using the actual interval Δ*t* (h) from the end of surgery to the first postoperative CT:

$$\mathrm{Brain}\;\mathrm{re}\text{-}\mathrm{expansion}\;\mathrm{rate}(\text{\%}/24\;h)=[(V_{\mathrm{pre}}-V_{\mathrm{post}})/V_{\mathrm{pre}}]\times (24/\Delta t)\times 100.$$

Higher values indicated a faster mean early re-expansion rate; negative values were retained when *V*_post_ exceeded *V*_pre_. For the fluid-only analysis, residual fluid volume replaced *V*_post_. The primary models reported effects per 10-percentage-point/24 h increase, and quartile groups were used for descriptive stratification. Because the measure is standardized to 24 h using the actual imaging interval, its numerical value is not necessarily bounded by 100%/24 h when that interval is shorter than 24 h.

Images from 57 randomly selected patients were independently remeasured by another neuroradiologist who was blinded to clinical outcomes and the initial measurements. Interobserver agreement was assessed using a two-way random-effects, absolute-agreement, single-measurement intraclass correlation coefficient (ICC), denoted ICC(2,1) (18). The 95% confidence interval (CI) for each ICC was estimated using the percentile method with 2,000 paired bootstrap resamples. ICCs were calculated from the two independent measurements before joint review. If the absolute difference between the two measurements of either volume exceeded 10% of their mean, the measurements were jointly reviewed to determine the final value used in the primary analysis.

### 2.5 Outcome definition and landmark follow-up

Completion of the first postoperative CT was defined as the landmark, with follow-up continuing until the first symptomatic recurrence, death before recurrence, or postoperative day 90 after the index operation, whichever occurred first. Time was measured in postoperative days, and patients entered the risk set at their actual CT time. Endpoint status was classified into three categories: symptomatic recurrence, death before recurrence, and survival without recurrence. In the cause-specific Cox and Kaplan–Meier (KM) analyses, the eight patients who died before recurrence were censored on the date of death, and the 231 patients who remained alive without recurrence were administratively censored on postoperative day 90. In the competing-risk analyses, symptomatic recurrence, death before recurrence, and administrative censoring were coded as 1, 2, and 0, respectively.

The primary outcome was symptomatic recurrence on the operated side requiring reintervention within 90 days after surgery and meeting all three of the following criteria: recurrence or worsening of existing neurological symptoms, or a new focal neurological deficit; reformation or enlargement of a subdural collection on the operated side on follow-up CT compared with the first postoperative CT; and consequent repeat burr-hole drainage, craniotomy for hematoma evacuation, or salvage middle meningeal artery embolization. The adjunctive use of middle meningeal artery embolization in recurrent CSDH and its therapeutic role in patients with particular surgical risks have been addressed in a multidisciplinary consensus statement (19). Reinterventions were classified by the first procedure actually performed. Radiographic progression without reintervention was recorded separately. For simultaneous bilateral surgery, the first recurrence on either operated side was counted as one patient-level event; progression on the unoperated side in patients with bilateral disease treated unilaterally was not included in the primary outcome.

Outcomes were ascertained from our hospital's inpatient, outpatient, imaging, and reintervention records. For 32 patients who did not undergo follow-up at our hospital, outcomes were verified by telephone together with external imaging and intervention records. Two neurosurgeons blinded to brain re-expansion measurements independently adjudicated outcomes, with disagreements resolved by a third physician. The timing of anticoagulation resumption is a temporal factor requiring evaluation in perioperative management (20). Antithrombotic drug class, last administration, and resumption time were verified against medication orders, discharge summaries, and follow-up records. The preoperative interruption interval was defined as the time from the last dose to the start of surgery, and the postoperative resumption interval as the time from the end of surgery to the first resumed dose; both were measured in days.

### 2.6 Statistical analysis

Approximately symmetrically distributed continuous variables were summarized as mean ± standard deviation, and skewed or ordinal variables as median [25th percentile, 75th percentile]. Between-group comparisons used Welch's independent-samples *t*-test or the two-sided Mann–Whitney *U*-test, respectively. An exact Mann–Whitney *U*-test was used for the small anticoagulant subgroup; asymptotic rank tests accounted for tied ranks and included continuity correction. Categorical variables were expressed as counts (percentages) and compared using Pearson's *χ*^2^ test; its applicability was assessed according to the number and proportion of cells with low expected counts. Variables with multiple categories were assessed using an overall distribution test. Descriptive comparisons were grouped by whether symptomatic recurrence was recorded; the group without recorded recurrence included patients who died before recurrence.

The Benjamini–Hochberg method was used to control the false discovery rate (FDR) across 38 exploratory comparisons of clinical, imaging, and perioperative characteristics; both raw *P* values and adjusted *P*_FDR_ values were reported. Each comparison was counted only once if repeated in a main table, and between-group comparisons were not used to select regression covariates. Statistical significance was defined as *P*_FDR_ < 0.05 for exploratory comparisons and *P* < 0.05 for other analyses.

Cause-specific Cox proportional hazards regression was used as the primary association framework rather than Fine–Gray regression because the primary estimand was the instantaneous recurrence hazard among patients who remained alive and recurrence-free. Fine–Gray regression was retained as a competing-risk sensitivity analysis to estimate the subdistribution hazard and 90-day cumulative incidence of symptomatic recurrence in the presence of competing death. Time was measured in postoperative days, and patients entered the risk set at the first postoperative CT. In the cause-specific analyses, the eight patients who died before recurrence were censored on the date of death, whereas the 231 patients who remained alive without recurrence were administratively censored on postoperative day 90. Adjustment factors were age, preoperative antithrombotic use, bilateral disease, moderate-to-severe brain atrophy, and preoperative hematoma volume. To address nonproportional effects identified by proportional hazards testing, the primary model was jointly stratified by the four combinations of brain atrophy and antithrombotic use, allowing the baseline hazard to vary across strata. Age, bilateral disease, and preoperative hematoma volume were included as regression covariates. The cause-specific hazard ratio (HR), 95% confidence interval (CI), Wald *z* statistic, and two-sided *P* value were reported per 10-percentage-point/24 h increase in brain re-expansion rate. Tied event times were handled using the Breslow method.

The cumulative incidence of symptomatic recurrence was estimated using the Aalen–Johansen estimator, with death before recurrence treated as a competing event. Separately, 1 − Kaplan–Meier was used to describe net recurrence with deaths censored, and the log-rank test compared the death-censored curves across quartile groups. In both analyses, risk-set entry occurred at the first postoperative CT. Kaplan–Meier CIs used Greenwood's variance and the complementary log–log transformation. All included patients had completed landmark CT before the first endpoint event, and there was no other censoring before postoperative day 90. Consequently, the Aalen–Johansen estimate at the fixed 90-day time point equaled the number of recurrences divided by the number of patients in the corresponding group; CIs at this time point were calculated using the Wilson method.

Restricted cubic splines were used to evaluate nonlinearity in the association between brain re-expansion rate and recurrence hazard, with knots at the 10th, 50th, and 90th percentiles and the median as the reference. The spline model was compared with the clinical base model and the linear-exposure model using likelihood ratio tests to assess the overall association and nonlinear component, respectively. The proportional hazards assumption was assessed using efficient score tests for interactions between each covariate and the centered natural logarithm of postoperative time, accounting for the remaining coefficients as nuisance parameters. Variable-specific and global test results were reported for the unstratified model, the model stratified by brain atrophy alone, and the jointly stratified model. The proportional subdistribution hazards assumption for the Fine–Gray model was tested using the corresponding extended risk set.

Sensitivity analyses included jointly stratified Fine–Gray regression, unstratified cause-specific Cox regression, re-expansion calculated using residual fluid alone, the relative volume reduction without time standardization with additional adjustment for the CT interval, total postoperative residual volume, absolute brain re-expansion rate [(*V*_pre_ − *V*_post_) × 24/Δ*t*], exclusion of patients with bilateral disease treated unilaterally, and binary logistic regression for the 90-day outcome. All models retained the same five adjustment factors. Except for the unstratified model, survival models used the same four-stratum structure, represented by three indicator variables in the logistic model. Models of residual volume and absolute re-expansion rate retained adjustment for preoperative volume. Fine–Gray models reported the subdistribution hazard ratio (sHR) with patient-level robust standard errors.

In the Fine–Gray analyses, deaths before recurrence were treated as competing events and retained in the corresponding subdistribution risk set. All patients had completed CT before the first endpoint event, and no other censoring occurred before postoperative day 90; therefore, censoring-survival weights at recurrence times were all 1. The binary logistic model evaluated whether symptomatic recurrence occurred before death within 90 days and reported the odds ratio (OR); patients who died before recurrence were coded as 0.

The clinical base model included age, bilateral disease, preoperative hematoma volume, and the joint stratification structure for brain atrophy and antithrombotic use; the full model additionally included brain re-expansion rate. Cause-specific Cox models were assessed using the stratified Harrell concordance index (C-index), calculated by separately summing the numbers of concordant pairs and all comparable patient pairs within each stratum; linear predictors were not compared across strata. Fine–Gray models calculated individual 90-day recurrence probabilities from the baseline cumulative subdistribution hazard for the patient's stratum and the regression coefficients. Predicted probabilities for all patients were used to calculate the fixed-time area under the receiver operating characteristic curve (AUC) and Brier score. The outcome indicator was 1 for the 47 patients with recurrence and 0 for the eight patients who died before recurrence and the 231 patients who remained alive without recurrence.

The recalibration intercept and slope were jointly estimated in a single logistic model, with recurrence by day 90 as the dependent variable and the logit of the predicted probability as the independent variable. Grouped calibration compared mean predicted probabilities with Aalen–Johansen observed cumulative incidence across tertiles of predicted risk from the full Fine–Gray model, with Wilson 95% CIs. Internal validation used 1,000 patient-level bootstrap resamples. The joint stratification structure was held fixed, and coefficients and stratum-specific baseline hazards for both the base and full models were refitted in each resample. CIs for single-model discrimination used the percentile method; CIs and *P* values for between-model differences used a normal approximation based on the paired bootstrap standard error. Optimism was estimated from performance differences between each bootstrap training sample and the original analysis sample, and optimism-corrected discrimination, Brier scores, and jointly estimated recalibration parameters were reported.

Between-group comparisons relating mobilization timing to drainage volume and brain re-expansion rate used the Mann–Whitney *U*-test. Brain re-expansion rate was analyzed using median regression (*τ* = 0.50) with asymptotic robust standard errors. Drainage volume was natural-log-transformed and analyzed using multivariable linear regression with the heteroskedasticity-consistent covariance estimator type 3 (HC3); exponentiated coefficients represented geometric mean ratios. Both models adjusted for age, admission Markwalder grade, anesthetic technique, bilateral disease, moderate-to-severe brain atrophy, and preoperative hematoma volume. The mobilization-related Cox model retained the joint stratification structure and covariates of the primary model and additionally included mobilization timing. Risk-set entry occurred at the later of the first postoperative CT or 24 h after surgery, and deaths before recurrence were censored on the date of death. Mobilization-related comparisons were not included in the 38 exploratory comparisons described above.

Data on the primary exposure, outcome, and model variables were complete. Missing descriptive variables were handled using available-case analysis. All statistical analyses were performed using Python 3.13.5. Quantiles of continuous variables were calculated using linear interpolation. Analyses were based on unrounded patient-level measurements, with decimal places standardized only for reporting. The *t*-test, Mann–Whitney *U*-test, and Wald tests were two-sided.

## 3 Results

### 3.1 Participants and data completeness

During the study period, 356 patients underwent surgery for CSDH. Patients were sequentially excluded for previous CSDH surgery (*n* = 11), acute or subacute subdural hematoma or isolated subdural hygroma (*n* = 8), another surgical procedure (*n* = 7), concomitant lesions affecting intracranial volumetric assessment (*n* = 6), ineligible preoperative CT or original DICOM data (*n* = 13), first postoperative CT outside the 22–26 h window or unusable imaging (*n* = 15), incomplete drainage records or primary-model variables (*n* = 4), repeat surgery before the landmark (*n* = 3), or an unverifiable 90-day outcome (*n* = 3). The final sample comprised 286 patients: 47 developed symptomatic recurrence, eight died before recurrence, and 231 remained alive without recurrence through postoperative day 90. Outcomes were verified from our hospital's records for 254 patients and by telephone together with external records for 32 patients (Figure 1).

**Figure 1:**
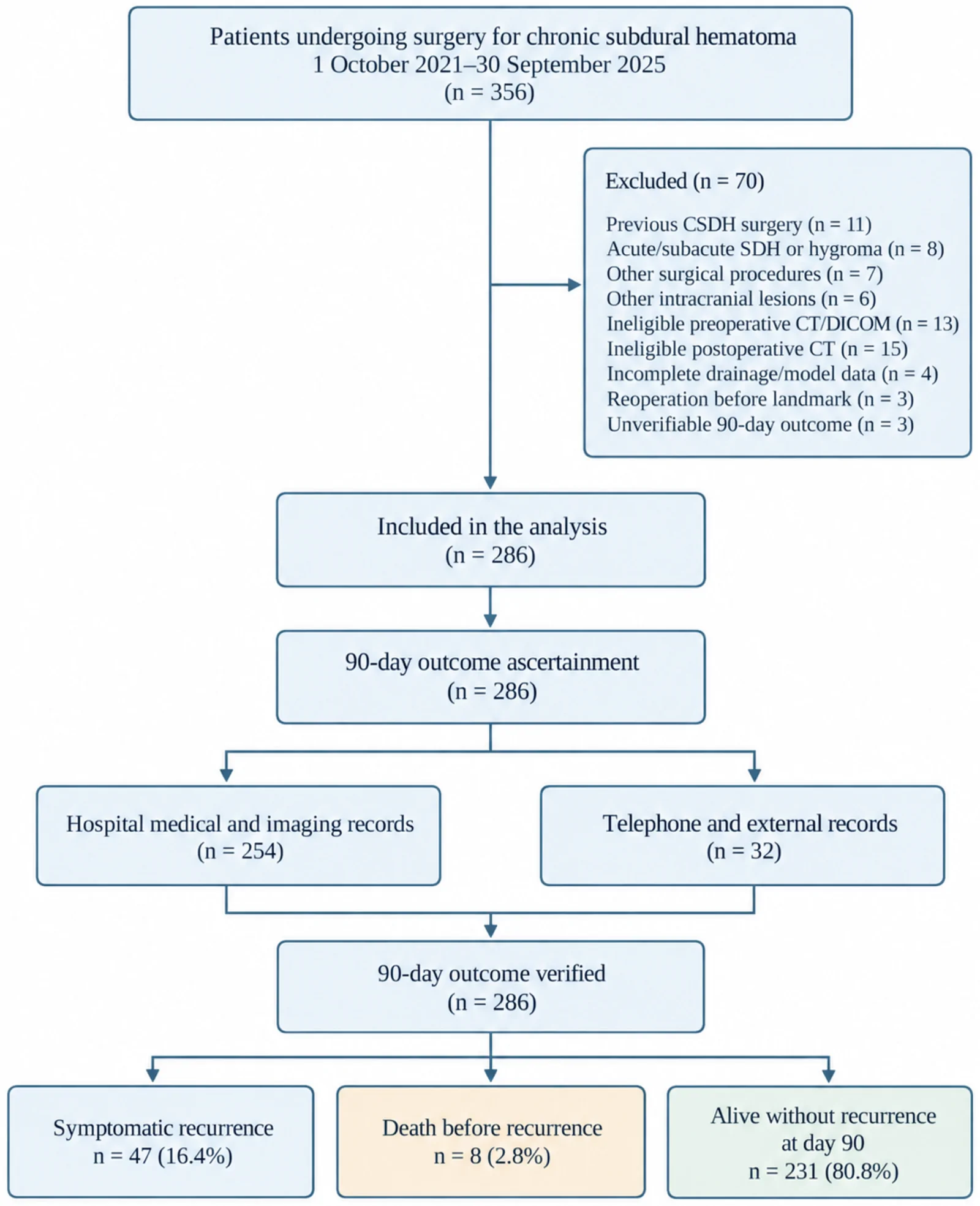
Participant selection and 90-day outcome ascertainment. A total of 356 patients who underwent surgery for chronic subdural hematoma (CSDH) between October 1, 2021, and September 30, 2025, were screened. Seventy patients were excluded according to nine mutually exclusive reasons, leaving 286 patients for analysis. Outcomes were ascertained from hospital records for 254 patients and by telephone together with external records for 32 patients. The 90-day outcomes comprised symptomatic recurrence in 47 patients (16.4%), death before recurrence in eight (2.8%), and survival without recurrence in 231 (80.8%). Computed tomography (CT); subdural hematoma (SDH); Digital Imaging and Communications in Medicine (DICOM). *n* denotes the number of patients.

Primary-model variables were complete. Nineteen patients had missing descriptive data: body mass index in five, recent head trauma in three, platelet count in four, international normalized ratio in three, and activated partial thromboplastin time in four.

### 3.2 Clinical characteristics

Mean age was 76.7 years in the recurrence group and 74.0 years in the group without recorded recurrence; preoperative antithrombotic use was 38.3% and 25.5%, respectively. After adjustment for multiple testing across the 38 exploratory comparisons, no clinical characteristic differed significantly between groups (all *P*_FDR_ > 0.05) (Table 1; Supplementary Table S2).

**Table 1:** Main clinical, imaging, and perioperative characteristics.

Variable	Overall	No recorded symptomatic recurrence (*n* = 239)	Symptomatic recurrence (*n* = 47)	Test statistic	Raw *P*	*P*_FDR_
Age, years	74.4 ± 9.2	74.0 ± 9.3	76.7 ± 8.1	*t*(72.0) = −2.036	0.045	0.195
Preoperative antithrombotic use, *n* (%)	79 (27.6)	61 (25.5)	18 (38.3)	*χ*^2^ (1) = 3.206	0.073	0.232
Hematoma laterality, *n* (%)	—	—	—	*χ*^2^ (2) = 4.225	0.121	0.302
Left	100 (35.0)	88 (36.8)	12 (25.5)	—	—	—
Right	104 (36.4)	88 (36.8)	16 (34.0)	—	—	—
Bilateral	82 (28.7)	63 (26.4)	19 (40.4)	—	—	—
Preoperative hematoma volume on the operated side, mL	114.2 ± 33.0	112.4 ± 32.3	123.6 ± 34.8	*t*(62.6) = −2.034	0.046	0.195
Internal hematoma architecture, *n* (%)	—	—	—	*χ*^2^ (3) = 12.099	0.007	0.038
Homogeneous	111 (38.8)	100 (41.8)	11 (23.4)	—	—	—
Laminar	62 (21.7)	52 (21.8)	10 (21.3)	—	—	—
Separated	68 (23.8)	48 (20.1)	20 (42.6)	—	—	—
Trabecular	45 (15.7)	39 (16.3)	6 (12.8)	—	—	—
Moderate-to-severe brain atrophy, *n* (%)	108 (37.8)	81 (33.9)	27 (57.4)	*χ*^2^ (1) = 9.273	0.002	0.018
Drainage volume during postoperative hours 0–24, mL	126 [93, 174]	129 [100, 176]	94 [73, 153]	*U* = 7,112.0	0.004	0.025

Data are presented as mean ± standard deviation, median [25th percentile, 75th percentile], or count (percentage). The group without recorded symptomatic recurrence included eight patients who died before recurrence and 231 who remained alive without recurrence. The symbols *t*, *U*, and *χ*^2^ denote the Welch independent-samples *t* statistic, Mann–Whitney *U* statistic, and Pearson chi-square statistic, respectively; values in parentheses after the statistics indicate degrees of freedom. *P* denotes the *P* value of the corresponding test. The false discovery rate (FDR) was controlled using the Benjamini–Hochberg method; *P*_FDR_ denotes the adjusted *P* value across 38 exploratory comparisons, with each comparison counted once. For variables with multiple categories, *P* values correspond to overall distribution tests. *n*, number of patients with the characteristic or the indicated analysis sample size; —, not applicable.

### 3.3 Preoperative imaging and perioperative characteristics

The overall distribution of internal hematoma architecture differed between the recurrence group and the group without recorded symptomatic recurrence, with separated-type hematomas accounting for 42.6% and 20.1%, respectively (overall *P*_FDR_ = 0.038). Moderate-to-severe brain atrophy was more common and drainage volume during the first 0–24 h was lower in the recurrence group (*P*_FDR_ = 0.018 and 0.025, respectively). No other preoperative imaging or perioperative characteristic differed significantly after adjustment for multiple testing (Table 1; Supplementary Table S3).

Brain re-expansion rate and drainage volume during the first 0–24 h did not differ significantly between the early and delayed mobilization groups. The adjusted median difference in brain re-expansion rate was 5.1 percentage points/24 h (95% CI, −2.2 to 12.3; *P* = 0.172), and the geometric mean ratio for drainage volume was 0.97 (95% CI, 0.85–1.11; *P* = 0.637). In the mobilization-related Cox model, the HR for early mobilization was 1.31 (95% CI, 0.62–2.78; *P* = 0.485), and the HR per 10-percentage-point/24 h increase in brain re-expansion rate was 0.74 (95% CI, 0.64–0.85; *P* < 0.001) (Supplementary Table S4).

### 3.4 Early postoperative brain re-expansion measures and measurement agreement

Total postoperative residual volume and residual fluid volume were greater, and brain re-expansion rates calculated with or without air were lower, in the recurrence group (all *P*_FDR_ < 0.01). The interval to the first postoperative CT and air volume did not differ significantly between groups (both *P*_FDR_ > 0.05). ICCs for preoperative volume, total postoperative residual volume, and brain re-expansion rate were 0.996, 0.998, and 0.993, respectively. Three patients had negative brain re-expansion rates. In five patients, the relative absolute difference between paired volume measurements exceeded 10%, and final values were determined by joint review (Table 2; Figure 2).

**Table 2:** Early postoperative imaging measurements and interobserver agreement.

Between-group comparisons						
Variable	Overall	No recorded symptomatic recurrence (*n* = 239)	Symptomatic recurrence (*n* = 47)	Test statistic	Raw *P*	*P*_FDR_
Interval from surgery completion to postoperative CT, h	24.0 ± 0.9	24.0 ± 0.9	23.9 ± 1.0	*t*(62.9) = 0.650	0.518	0.679
Total postoperative residual subdural volume, mL	49.6 [32.2, 71.7]	46.8 [31.0, 64.8]	74.5 [49.2, 106.9]	*U* = 3,259.0	<0.001	<0.001
Subdural air volume, mL	9.5 [7.2, 13.3]	9.3 [6.9, 13.2]	10.0 [8.6, 13.6]	*U* = 4,631.0	0.057	0.218
Postoperative residual fluid volume, mL	38.4 [21.2, 60.6]	35.9 [20.5, 55.2]	62.8 [38.8, 94.6]	*U* = 3,434.0	<0.001	<0.001
24 h brain re-expansion rate, %/24 h	54.2 [39.7, 67.2]	56.8 [43.4, 68.8]	40.8 [26.3, 54.3]	*U* = 7,884.0	<0.001	<0.001
Brain re-expansion rate excluding air, %/24 h	65.0 [49.7, 78.1]	66.8 [52.0, 79.7]	50.8 [33.4, 69.8]	*U* = 7,584.0	<0.001	0.001
Interobserver agreement						
Measurement	Paired sample size	ICC(2,1)	95% CI			
Preoperative hematoma volume	57	0.996	0.994–0.998			
Total postoperative residual subdural volume	57	0.998	0.996–0.999			
24 h brain re-expansion rate	57	0.993	0.987–0.996			

Data are presented as mean ± standard deviation or median [25th percentile, 75th percentile]. The symbol *t* denotes the Welch independent-samples *t* statistic, with degrees of freedom in parentheses; *U* denotes the Mann–Whitney *U* statistic. *P* denotes a two-sided *P* value; *P*_FDR_ denotes the Benjamini–Hochberg-adjusted *P* value across 38 comparisons, where FDR denotes the false discovery rate. CT, computed tomography. ICC, intraclass correlation coefficient; ICC(2,1) denotes a two-way random-effects, absolute-agreement, single-measurement model based on 57 independent paired measurements obtained before joint review. CI, confidence interval, estimated using 2,000 paired bootstrap resamples. The group without recorded recurrence included eight patients who died before recurrence and 231 who remained alive without recurrence. *n*, number of patients; —, not applicable.

**Figure 2:**
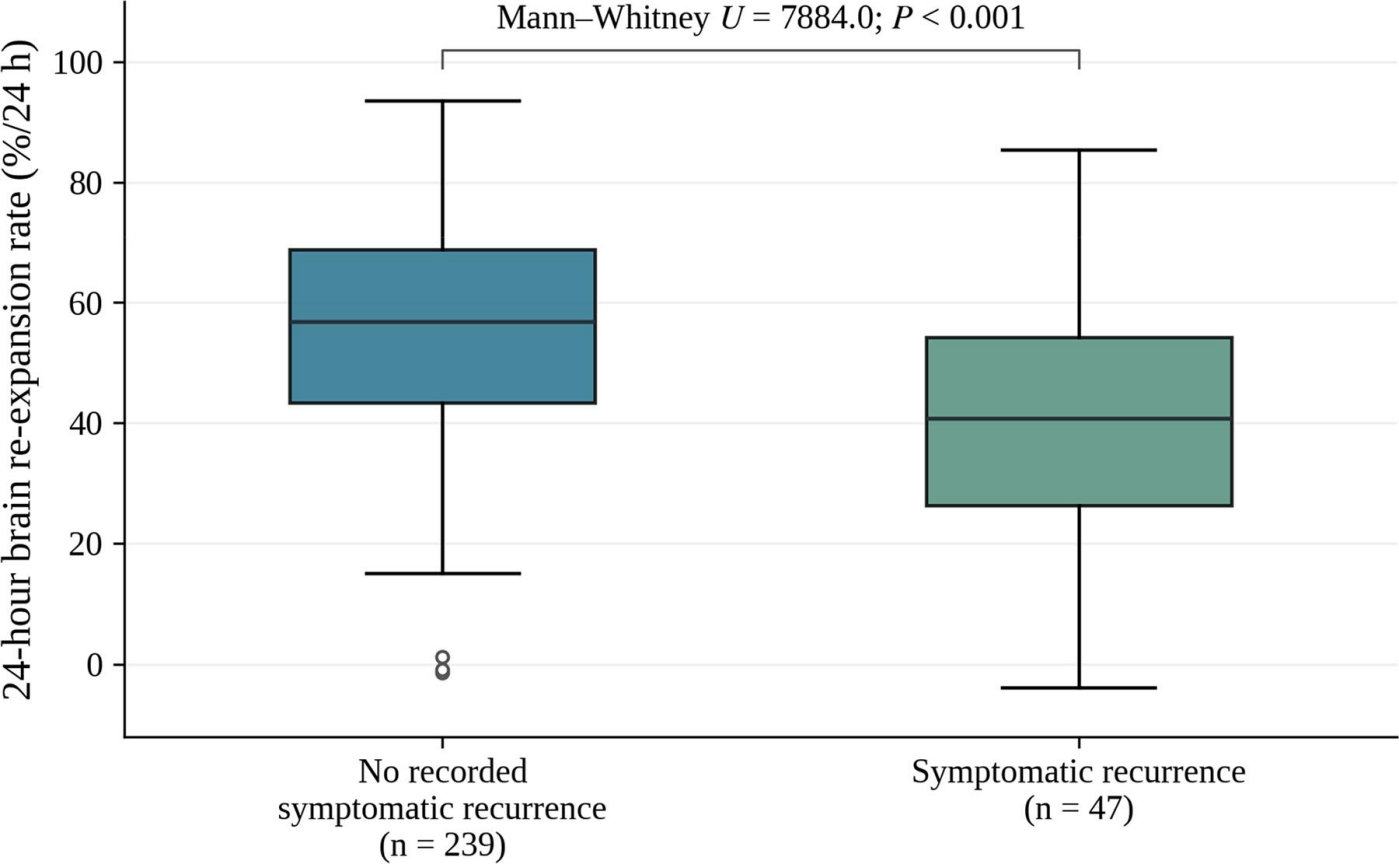
Distribution of the 24 h postoperative brain re-expansion rate by symptomatic recurrence status. The upper and lower edges of each box represent the 75th and 25th percentiles, respectively, and the horizontal line within the box indicates the median. Whiskers extend to the most extreme observations within 1.5 times the interquartile range from the corresponding quartile; open circles indicate observations beyond this range. The group without recorded symptomatic recurrence comprised 239 patients, including eight who died before recurrence; the symptomatic recurrence group comprised 47 patients. The two-sided Mann–Whitney *U*-test yielded *U* = 7,884.0 and *P* < 0.001. After Benjamini–Hochberg adjustment for the false discovery rate (FDR) across 38 exploratory comparisons, *P*_FDR_ < 0.001. *U* denotes the test statistic, *P* the two-sided *P* value, and *n* the number of patients in each group.

### 3.5 Ninety-day postoperative outcomes and reinterventions

By postoperative day 90, 47 patients (16.4%) had developed symptomatic recurrence, eight (2.8%) had died before recurrence, and 231 (80.8%) remained alive without recurrence. Total follow-up was 22,596.1 person-days, with a mean of 79.0 days. The interval from the CT landmark to recurrence was 32.9 [19.2, 50.1] days. First reinterventions comprised repeat burr-hole drainage in 39 patients, craniotomy for hematoma evacuation in five, and salvage middle meningeal artery embolization in three. Nine additional patients had radiographic progression without reintervention: seven had no new or worsening neurological symptoms, and two had mild headache that resolved with conservative treatment (Table 3).

**Table 3:** Ninety-day postoperative outcomes and reinterventions.

90-day outcome or management	Value	Additional information
Symptomatic recurrence	47/286 (16.4%)	Primary outcome
Death before recurrence	8/286 (2.8%)	Competing event; censored on the date of death in cause-specific analyses
Alive without recurrence through postoperative day 90	231/286 (80.8%)	Administratively censored on postoperative day 90
No recorded symptomatic recurrence (total)	239/286 (83.6%)	Sum of 8 deaths before recurrence and 231 administrative censorings
Radiographic progression alone, without reintervention	9/286 (3.1%)	Included among the 239 patients without the recorded primary outcome
Of these: no new or worsening neurological symptoms	7/9 (77.8%)	Follow-up and conservative treatment
Of these: mild headache that subsequently resolved	2/9 (22.2%)	Conservative treatment
Repeat burr-hole drainage	39/47 (83.0%)	First reintervention
Craniotomy for hematoma evacuation	5/47 (10.6%)	First reintervention
Salvage middle meningeal artery embolization	3/47 (6.4%)	First reintervention
Interval from CT landmark to recurrence, days	32.9 [19.2, 50.1]	47 patients with recurrence
Total follow-up, person-days	22,596.1	Ended at recurrence, death, or postoperative day 90
Mean follow-up, days	79.0	Total follow-up divided by 286

The first three endpoint categories are mutually exclusive and sum to 286 patients. The group without recorded symptomatic recurrence comprises eight patients who died before recurrence and 231 who remained alive without recurrence; radiographic progression alone is a descriptive subgroup of these patients. Reinterventions are classified according to the first procedure performed, with the 47 patients with recurrence as the denominator. The interval to recurrence is expressed as median [25th percentile, 75th percentile]. Total follow-up is the sum of individual intervals from the first postoperative computed tomography (CT) to first recurrence, death before recurrence, or postoperative day 90; mean follow-up is total person-time divided by 286. Percentage denominators are 286, 9, or 47, as appropriate.

### 3.6 Cumulative recurrence by quartiles of brain re-expansion rate

The first through fourth quartile groups of brain re-expansion rate comprised 72, 71, 71, and 72 patients, with 22, 13, seven, and five symptomatic recurrences, respectively. The 90-day cumulative incidences accounting for competing death were 30.6%, 18.3%, 9.9%, and 6.9%; the corresponding net recurrence estimates with deaths censored were 31.0%, 18.3%, 9.9%, and 7.0%. The death-censored curves differed significantly across groups (log-rank *χ*^2^ = 17.449, 3 degrees of freedom; *P* < 0.001) (Table 4; Figure 3).

**Table 4:** Ninety-day outcomes by quartiles of brain re-expansion rate.

Group	Range %/24 h	*n*	Recurrence	Death	Administrative censoring	Aalen–Johansen %(95% CI)	1 − KM %(95% CI)
First quartile	≤39.7	72	22	2	48	30.6 (21.1–42.0)	31.0 (21.6–43.2)
Second quartile	>39.7–54.2	71	13	1	57	18.3 (11.0–28.8)	18.3 (11.1–29.5)
Third quartile	>54.2–67.2	71	7	3	61	9.9 (4.9–19.0)	9.9 (4.9–19.7)
Fourth quartile	>67.2	72	5	2	65	6.9 (3.0–15.2)	7.0 (3.0–15.9)
Overall	—	286	47	8	231	16.4 (12.6–21.2)	16.6 (12.7–21.5)

Death denotes death before recurrence; administrative censoring denotes survival without symptomatic recurrence through postoperative day 90. In each row, the numbers of recurrences, deaths, and administrative censorings sum to the group size (*n*). The Aalen–Johansen estimate is the cumulative incidence of recurrence accounting for competing death; 1 − Kaplan–Meier (KM) is the net recurrence estimate with deaths censored. Confidence intervals (CIs) at the fixed 90-day time point were calculated using the Wilson method and Greenwood's variance with the complementary log–log transformation, respectively. The log-rank test compares death-censored KM curves; *χ*^2^ denotes its test statistic, the value in parentheses indicates degrees of freedom, and *P* denotes the log-rank *P* value. This test does not compare Aalen–Johansen curves. Grouping used unrounded brain re-expansion rates, with cutoffs expressed in %/24 h. —, not applicable.

**Figure 3:**
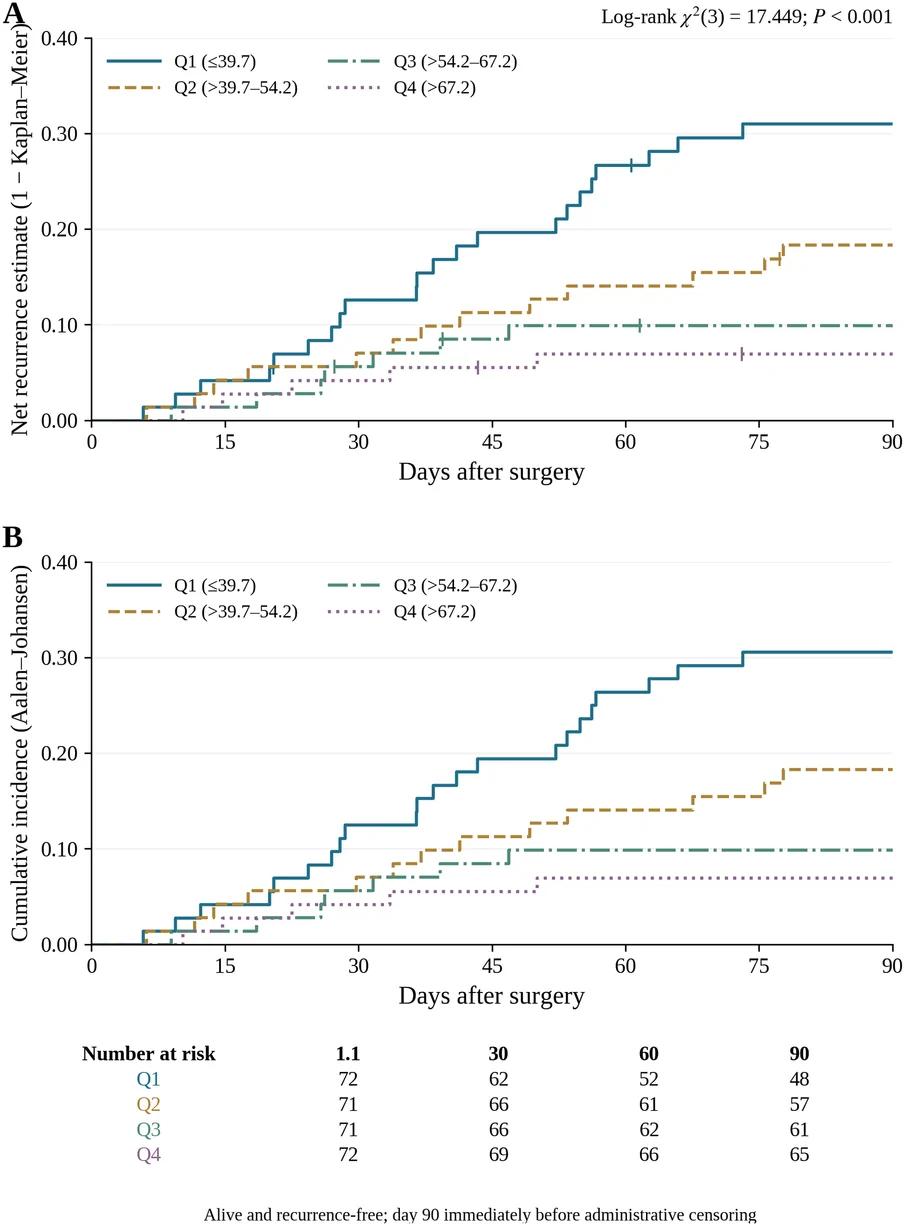
Estimates of symptomatic recurrence by quartiles of the 24 h postoperative brain re-expansion rate. **(A)** Net recurrence estimated as 1 − Kaplan–Meier, with deaths before recurrence censored on the date of death; tick marks indicate censoring due to death. **(B)** Aalen–Johansen cumulative incidence, with death before recurrence treated as a competing event. Time is measured in postoperative days, and patients enter the risk set at the first postoperative computed tomography (CT). The shared risk table shows patients who remained alive and recurrence-free at days 1.1, 30, 60, and 90; the day-90 counts are immediately before administrative censoring. Q1–Q4 denote the first through fourth quartile groups, with cutoffs of 39.7, 54.2, and 67.2%/24 h. The log-rank *χ*^2^(3) = 17.449, *P* < 0.001, applies only to the death-censored curves in panel A. *χ*^2^, test statistic, with degrees of freedom in parentheses; *P*, log-rank *P* value.

### 3.7 Cox regression and restricted cubic spline analyses

In the unadjusted model, the cause-specific HR per 10-percentage-point/24 h increase in brain re-expansion rate was 0.72 (95% CI, 0.63–0.83; *P* < 0.001). After adjustment with joint stratification, the HR was 0.74 (95% CI, 0.64–0.85; *P* < 0.001). The HRs for each 10-year increase in age and for bilateral disease were 1.46 and 2.12, respectively (*P* = 0.018 for both), whereas the association with preoperative hematoma volume was not statistically significant (*P* = 0.078). Brain atrophy and antithrombotic use were stratification factors, and common HRs were not estimated for them (Table 5).

**Table 5:** Cause-specific Cox regression analysis of the 24 h brain re-expansion rate and 90-day recurrence.

Variable	Unadjusted HR (95% CI)	*z*	*P*	Stratified adjusted HR (95% CI)	*z*	*P*
Brain re-expansion rate, per 10-percentage-point/24 h increase	0.72 (0.63–0.83)	−4.650	<0.001	0.74 (0.64–0.85)	−4.147	<0.001
Age, per 10-year increase	—	—	—	1.46 (1.07–2.01)	2.369	0.018
Bilateral disease	—	—	—	2.12 (1.14–3.95)	2.362	0.018
Preoperative hematoma volume, per 10-mL increase	—	—	—	1.09 (0.99–1.20)	1.762	0.078
Preoperative antithrombotic use	—	—	—	Joint stratification factor	—	—
Moderate-to-severe brain atrophy	—	—	—	Joint stratification factor	—	—

HR, cause-specific hazard ratio; CI, confidence interval; *z*, Wald statistic; *P*, two-sided Wald test *P* value. The unadjusted model includes only brain re-expansion rate. The adjusted model is stratified by the four combinations of moderate-to-severe brain atrophy and preoperative antithrombotic use and includes age, bilateral disease, and preoperative hematoma volume. Common HRs are not estimated for stratification factors. Patients enter the risk set at the first postoperative computed tomography (CT); the eight deaths before recurrence are censored on the date of death, and the 231 patients who remained alive without recurrence are administratively censored on postoperative day 90. The effect of brain re-expansion rate is expressed per 10-percentage-point/24 h increase; unilateral disease is the reference for bilateral disease. —, not estimated or not applicable.

The global proportional hazards test for the unstratified cause-specific Cox model yielded *P* = 0.026, and the variable-specific test for moderate-to-severe brain atrophy yielded *P* = 0.010. After stratification by brain atrophy alone, the test for antithrombotic use yielded *P* = 0.031. For the jointly stratified model, the global test yielded *χ*^2^ = 2.844 with 4 degrees of freedom (*P* = 0.584); variable-specific *P* values for brain re-expansion rate, age, bilateral disease, and preoperative volume were 0.264, 0.938, 0.653, and 0.214, respectively. The global test for the jointly stratified Fine–Gray model yielded *P* = 0.574. In the restricted cubic spline analysis, *P* < 0.001 for the overall association and *P* = 0.994 for nonlinearity (Figure 4; Supplementary Table S5).

**Figure 4:**
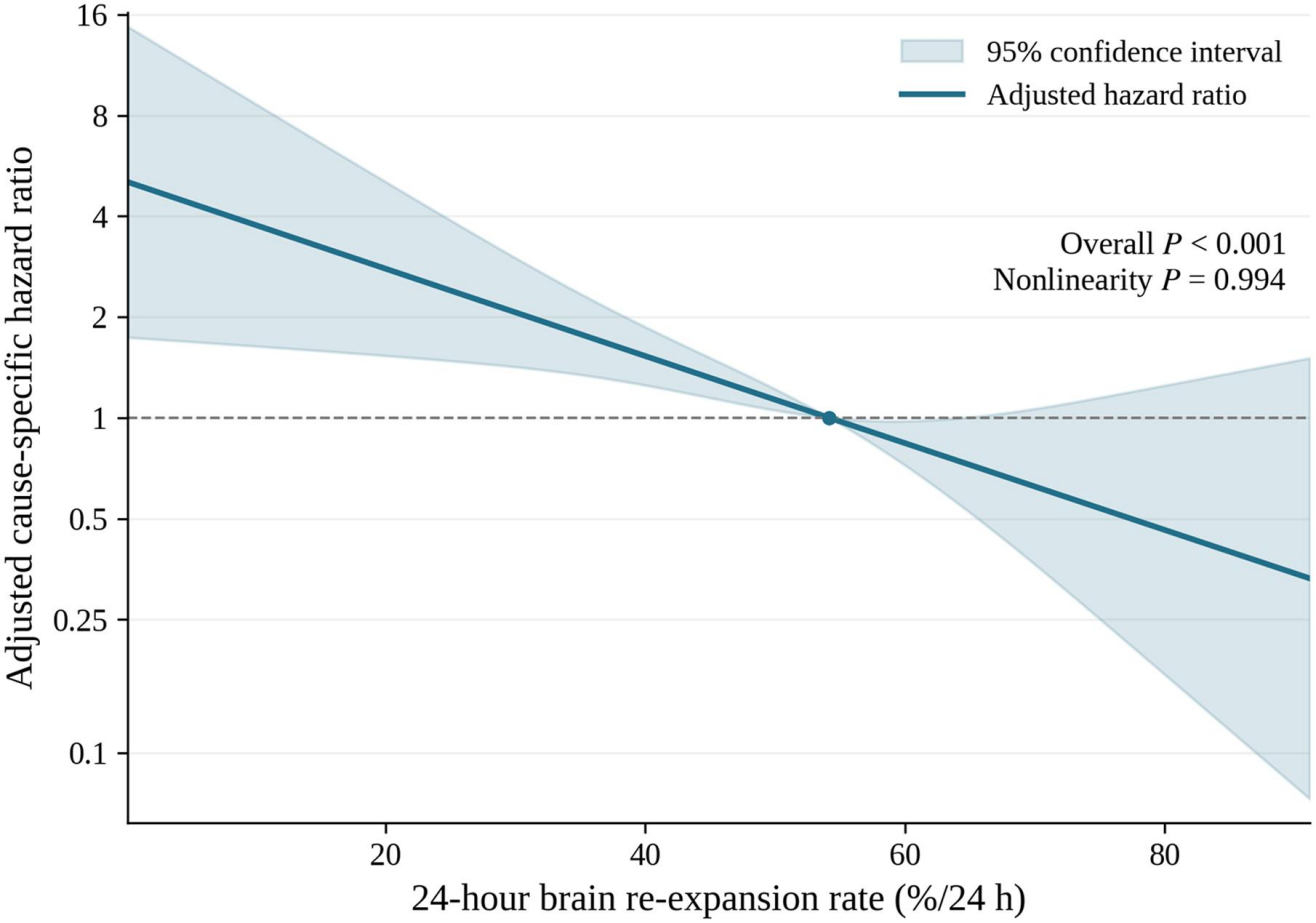
Restricted cubic spline analysis of the association between the 24 h postoperative brain re-expansion rate and the cause-specific hazard of 90-day symptomatic recurrence. The solid line represents the adjusted hazard ratio (HR), the shaded area the 95% confidence interval (CI), and the horizontal dashed line HR = 1. The reference is 54.2%/24 h, with knots at 26.2, 54.2, and 77.3%/24 h. The horizontal axis spans the first to the 99th percentile (0.15–91.18%/24 h). The model is jointly stratified by brain atrophy and antithrombotic use and adjusted for age, bilateral disease, and preoperative volume; deaths before recurrence are censored on the date of death. Overall association *P* < 0.001; nonlinearity *P* = 0.994. CIs are based on coefficient contrasts relative to the same reference and the covariance matrix. The vertical axis is logarithmic. *P*, likelihood ratio test *P* value.

### 3.8 Sensitivity analyses

In the jointly stratified Fine–Gray model, the sHR per 10-percentage-point/24 h increase in brain re-expansion rate was 0.74 (95% CI, 0.65–0.85; *P* < 0.001), consistent in direction with the primary cause-specific model. Associations were directionally consistent in the unstratified model, analyses using alternative re-expansion measures, the analysis excluding 24 patients with bilateral disease treated unilaterally, and the binary logistic analysis (all *P* < 0.001). Greater total postoperative residual volume was associated with a higher instantaneous recurrence hazard (*P* < 0.001) (Supplementary Table S6). In the full Fine–Gray model, the sHRs for age and bilateral disease were 1.47 and 2.13, respectively (*P* = 0.013 and 0.014); the association with preoperative volume was not statistically significant (*P* = 0.083). The four joint strata comprised 126, 52, 81, and 27 patients, respectively (Supplementary Table S7).

### 3.9 Model discrimination and calibration

The stratified C-index was 0.695 for the full model, compared with 0.657 for the base model, but the difference was not statistically significant (difference, 0.039; 95% CI, −0.015 to 0.092; *P* = 0.155). The 90-day AUC of the Fine–Gray model increased from 0.731 to 0.774 (difference, 0.042; 95% CI, 0.001–0.083; *P* = 0.043). The full model's optimism-corrected stratified C-index and AUC were 0.679 and 0.745, respectively, with a Brier score of 0.116 and recalibration intercept and slope of −0.12 and 0.89. Mean predicted probabilities in the low-, intermediate-, and high-risk groups were 4.4%, 10.9%, and 33.1%, respectively, compared with observed cumulative incidences of 6.3%, 9.5%, and 33.3% (Table 6; Figure 5).

**Table 6:** Ninety-day predictive performance of the clinical base and full models.

Model or measure	Apparent estimate (95% CI)	Optimism-corrected estimate or within-group outcomes	*P*
Cause-specific Cox: stratified C-index, base model	0.657 (0.582–0.764)	0.638	—
Cause-specific Cox: stratified C-index, full model	0.695 (0.613–0.801)	0.679	—
Stratified C-index difference: full−base	0.039 (−0.015–0.092)	—	0.155
Fine–Gray: 90-day AUC, base model	0.731 (0.672–0.826)	0.697	—
Fine–Gray: 90-day AUC, full model	0.774 (0.717–0.863)	0.745	—
90-day AUC difference: full−base	0.042 (0.001–0.083)	—	0.043
Fine–Gray: Brier score, base model	0.123	0.131	—
Fine–Gray: Brier score, full model	0.108	0.116	—
Fine–Gray: recalibration intercept, full model	0.07	−0.12	—
Fine–Gray: recalibration slope, full model	1.04	0.89	—
Low-risk tertile	Predicted 4.4%; observed 6.3%	*n* = 95; recurrences 6; deaths 2	—
Intermediate-risk tertile	Predicted 10.9%; observed 9.5%	*n* = 95; recurrences 9; deaths 5	—
High-risk tertile	Predicted 33.1%; observed 33.3%	*n* = 96; recurrences 32; deaths 1	—

The C-index is the stratified Harrell concordance index, calculated using only comparable patient pairs within the same joint stratum. AUC is the area under the receiver operating characteristic curve calculated from individual Fine–Gray-predicted 90-day probabilities. The Brier score is the mean squared difference between predicted probabilities and the 90-day recurrence indicator. The recalibration intercept and slope are jointly estimated in the same logistic model, with ideal values of 0 and 1, respectively. Both models are jointly stratified by brain atrophy and antithrombotic use and include age, bilateral disease, and preoperative volume; the full model additionally includes brain re-expansion rate. CI, confidence interval. Single-model discrimination CIs use the percentile method with 1,000 bootstrap resamples; CIs and two-sided *P* values for differences use a normal approximation based on the paired bootstrap standard error. The outcome indicator is 1 for the 47 patients with recurrence and 0 for the eight patients who died before recurrence and the 231 who remained alive without recurrence. Observed values are Aalen–Johansen estimates. *n*, group size; death, death before recurrence; —, not tested or not applicable.

**Figure 5:**
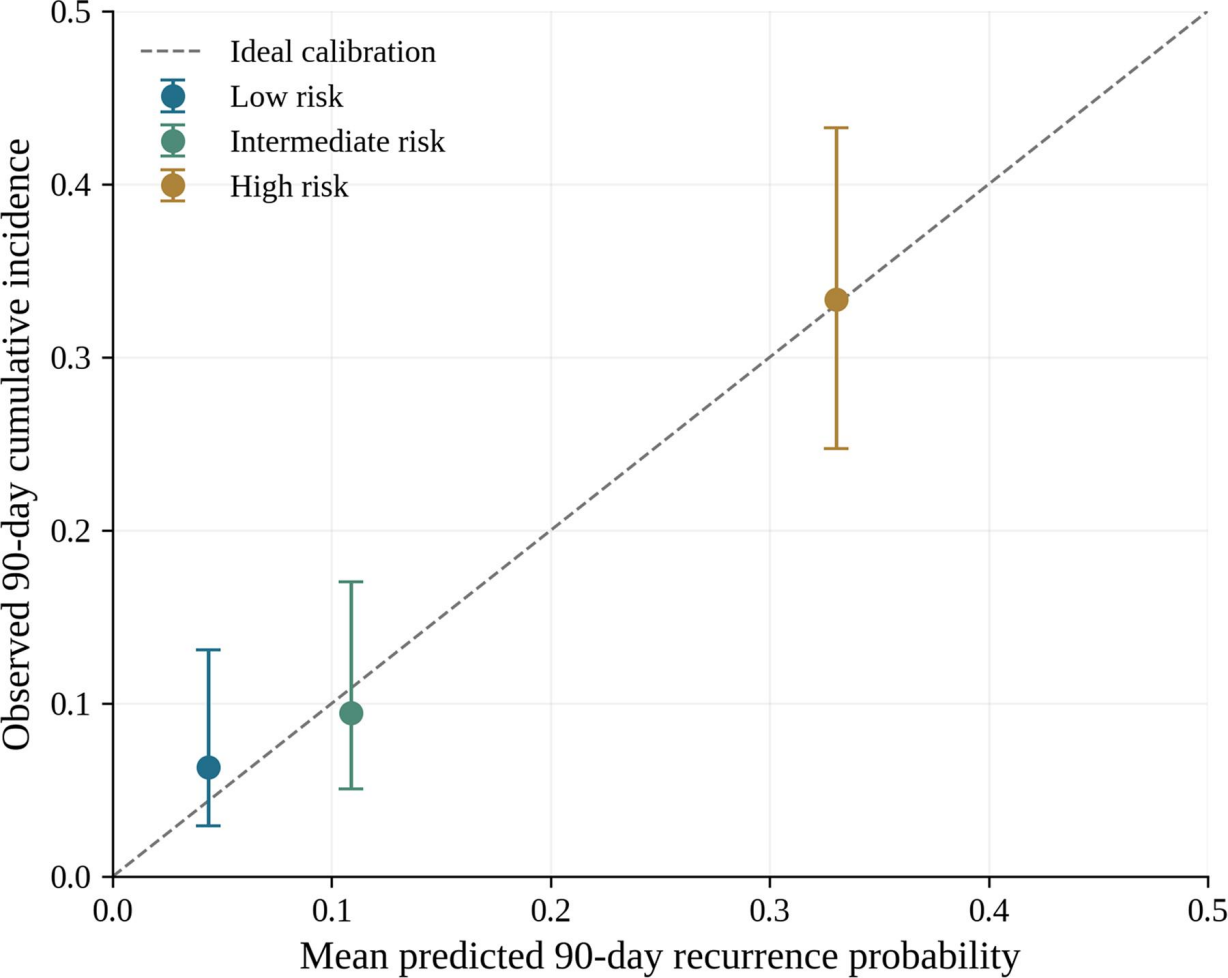
Grouped calibration of the full jointly stratified Fine–Gray model for 90-day symptomatic recurrence. The horizontal axis shows the mean predicted probability within each risk tertile, and the vertical axis shows the Aalen–Johansen observed cumulative incidence. Error bars indicate Wilson 95% confidence intervals (CIs), and the 45° dashed line indicates ideal calibration. The low-, intermediate-, and high-risk groups comprised 95, 95, and 96 patients, with six, nine, and 32 recurrences and two, five, and one deaths before recurrence, respectively. Mean predicted probabilities were 4.4%, 10.9%, and 33.1%, compared with observed values of 6.3%, 9.5%, and 33.3%. The figure shows apparent grouped calibration. After optimism correction using 1,000 bootstrap resamples, the jointly estimated recalibration intercept and slope were −0.12 and 0.89, respectively.

The full jointly stratified Fine–Gray model gives the probability of 90-day symptomatic recurrence as *F*_1_(90|X, s) = 1 − exp[−*H*_0*s*_(90) × exp(*η*)], where: *η* = −0.30081163 ×  (brain re-expansion rate/10) + 0.38287809 × [(age − 74)/10] + 0.75401077 × bilateral disease + 0.08495459 × [(preoperative hematoma volume − 114)/10].

*H*_0*s*_(90) is the baseline cumulative subdistribution hazard in the patient's joint stratum. For the 0/1 combinations of “moderate-to-severe brain atrophy, preoperative antithrombotic use,” *H*_0*s*_(90) is 0.29852759, 0.53857265, 0.58362526, and 1.53121662 in strata (0,0), (0,1), (1,0), and (1,1), respectively. Binary variables are coded 1 when present and 0 when absent. Brain re-expansion rate, age, and preoperative volume are expressed in %/24 h, years, and mL, respectively.

## 4 Discussion

This study showed that a lower first-postoperative-day brain re-expansion rate was associated with a higher cause-specific instantaneous hazard of 90-day symptomatic recurrence; the association remained directionally consistent in the subdistribution hazard analysis accounting for death before recurrence. Patients with recurrence had greater residual fluid and total residual volumes and lower re-expansion rates calculated with or without air. Outcome data after drainage likewise indicate that recurrence is an important component of postoperative assessment and that specific outcome definitions affect interpretation of recurrence proportions (21). The primary outcome in this study required the simultaneous presence of symptoms, radiographic progression, and reintervention, focusing its clinical interpretation on symptomatic recurrence requiring further treatment.

Brain atrophy may affect the capacity of brain tissue to reoccupy the subdural space after decompression; observations of brain ultrastructure and physical properties provide a biological basis for this interpretation (22). Moderate-to-severe brain atrophy was more common in the recurrence group, and its nonproportional effect was addressed through joint stratification. The overall distribution of internal hematoma architecture also differed between groups, with a greater proportion of separated-type hematomas in the recurrence group. Associations of cavity separation and neomembrane thickness with recurrence suggest that local structure may contribute to hematoma persistence (23). The first-day brain re-expansion rate integrates preoperative burden and the postoperative residual state, but its specific biological components require direct measurement.

Evidence linking postoperative drainage to lower recurrence risk supports attention to continued fluid evacuation after hematoma removal (24). Reports of valve-controlled drainage in selected patients provide feasibility data for an alternative drainage technique (25). In this study, patients with recurrence had lower drainage volumes during the first 24 h and greater residual fluid volumes, whereas between-group differences in air volume and drainage duration were not statistically significant after adjustment for multiple comparisons. These findings suggest that drainage volume, catheter status, and the residual cavity should be considered complementary observational measures; the observed associations do not directly establish a therapeutic effect of prolonged drainage.

Simultaneous and consecutive drainage of bilateral CSDH have not shown a significant difference in the treatment-success outcome evaluated (26). Bilateral disease was associated with higher recurrence risk in this study, which assessed disease distribution rather than drainage sequence. Both postoperative thromboembolic and hemorrhagic events in patients receiving anticoagulation may affect clinical outcomes (27). Preoperative antithrombotic use was incorporated into the joint stratification structure, and a constant HR was not estimated for this factor. Medication history, duration of interruption, and interval to resumption should nevertheless be evaluated separately.

Associations of the systemic immune-inflammation index and eosinophils with recurrence suggest that systemic immune status may contribute to hematoma persistence (28). The association between early spontaneous cessation of drainage and higher recurrence risk further emphasizes the relevance of postoperative fluid evacuation (29). The first-day re-expansion rate in this study combines relative volume reduction with the interval to imaging but does not directly measure membrane vascular permeability, inflammatory dynamics, or catheter flow profiles. It is therefore better interpreted as a composite imaging marker of the local postoperative state.

Intracranial pressure measurements during early mobilization indicate that coordination between changes in body position and drainage height warrants attention (30). Comparisons between head-of-bed elevation and the supine position have not demonstrated consistent differences in recurrence or major complications (31). In this study, the adjusted associations of mobilization timing with drainage volume, brain re-expansion rate, and recurrence were not statistically significant, whereas the association with brain re-expansion rate persisted after inclusion of mobilization timing. These results arose under the existing graded mobilization and closed-drainage management protocols. Mobilization timing, head-of-bed angle, and drainage height should be evaluated as distinct management factors.

Cognitive impairment may persist after CSDH treatment, and cognitive outcomes require independent assessment (32). Correlations between CT-estimated volume and intraoperatively evacuated volume indicate that volumetric information obtained at different stages is related, although measurement methods and the time points represented differ (33). This study restricted imaging to the first postoperative day and standardized for time, primarily characterizing the mean early volumetric re-expansion rate. Functional outcomes such as cognition, gait, and quality of life require follow-up using appropriate assessment instruments.

Further recurrence after middle meningeal artery embolization is associated with the preceding recurrence interval and the timing of embolization, suggesting that disease stage should inform interpretation of treatment outcomes (34). This study classified salvage embolization as one of the reinterventions performed when both symptoms and radiographic progression were present, with each category counted according to the first intervention. Brain re-expansion rate may assist in characterizing postoperative recurrence propensity, but the choice of reintervention still requires consideration of symptom evolution, serial imaging, and the patient's overall risk.

The cause-specific hazard describes the instantaneous recurrence rate among patients who remain alive and recurrence-free, whereas the subdistribution hazard is linked to the cumulative incidence function in the presence of competing death; the two estimands address different clinical questions (35). The direction and magnitude of the brain re-expansion rate effect were similar in the two jointly stratified models. Net recurrence estimates with deaths censored were slightly higher than cumulative incidences accounting for competing death, and the two measures should not be used interchangeably.

Prediction-model reporting should include discrimination, calibration, and the validation approach to distinguish apparent fit from generalizable performance (36). The difference in stratified C-index after adding brain re-expansion rate was not statistically significant, whereas the difference in 90-day AUC was significant. The full Fine–Gray model had a lower Brier score, but its corrected recalibration slope remained below 1. The increase in AUC reflects only a change in discrimination within the current data and does not establish clinical net benefit.

This study has several limitations. Retrospective data may be affected by selection bias, differences in treatment practice, and unmeasured confounding. The results primarily apply to patients with analyzable CT obtained 22–26 h after surgery who had not undergone reoperation before the landmark. Two imaging time points reflect only a mean re-expansion rate, and segmentation errors may remain at fluid and air boundaries. The 47 recurrence events limit model complexity, and the eight deaths before recurrence limit analyses of mortality-related heterogeneity. The joint stratification structure was selected on the basis of proportional hazards diagnostics in these data and held fixed during bootstrapping; internal validation therefore did not incorporate uncertainty from structure selection. The smallest stratum comprised only 27 patients, limiting the precision of its baseline hazard estimate. The primary outcome focused on symptomatic recurrence requiring reintervention. Clinical net benefit, cognition, gait, and quality of life require evaluation in independent data.

## 5 Conclusions

A faster 24 h brain re-expansion rate after burr-hole drainage of CSDH was associated with a lower cause-specific hazard of 90-day symptomatic recurrence. The association remained directionally consistent after accounting for competing death, joint stratification, and alternative exposure definitions. The incremental 90-day discrimination provided by this parameter requires external validation, and clinical application requires further evaluation of risk calibration and net benefit.

## Data Availability

The original contributions presented in the study are included in the article/Supplementary Material, further inquiries can be directed to the corresponding author/s.

## References

[1] El Rahal A Beck J Ahlborn P Bernasconi C Marbacher S Wanderer S. Incidence, therapy, and outcome in the management of chronic subdural hematoma in Switzerland: a population-based multicenter cohort study. Front Neurol. (2023) 14:1206996. 10.3389/fneur.2023.1206996 37780710 PMC10540067

[2] Hamou HA Clusmann H Schulz JB Wiesmann M Altiok E Höllig A. Chronic subdural hematoma: antithrombotics and thrombotic complications. Dtsch Arztebl Int. (2022) 119(12):208–13. 10.3238/arztebl.m2022.0144 35236548 PMC9277133

[3] Weigel R Schilling L Krauss JK. The pathophysiology of chronic subdural hematoma revisited: emphasis on aging processes as key factor. Geroscience. (2022) 44(3):1353–71. 10.1007/s11357-022-00570-y 35461468 PMC9213588

[4] Solou M Ydreos I Gavra M Papadopoulos EK Banos S Boviatsis EJ. Controversies in the surgical treatment of chronic subdural hematoma: a systematic scoping review. Diagnostics (Basel). (2022) 12(9):2060. 10.3390/diagnostics12092060 36140462 PMC9498240

[5] Rodriguez B Morgan I Young T Vlastos J Williams T Hrabarchuk EI. Surgical techniques for evacuation of chronic subdural hematoma: a mini-review. Front Neurol. (2023) 14:1086645. 10.3389/fneur.2023.1086645 37456631 PMC10338715

[6] Bartley A Bartek J Jakola AS Sundblom J Fält M Förander P. Effect of irrigation fluid temperature on recurrence in the evacuation of chronic subdural hematoma: a randomized clinical trial. JAMA Neurol. (2023) 80(1):58–63. 10.1001/jamaneurol.2022.4133 36409480 PMC9679960

[7] Lodewijkx R Foppen M Slot KAM Vandertop WP Verbaan D. Recurrent chronic subdural hematoma after burr-hole surgery and postoperative drainage: a systematic review and meta-analysis. Oper Neurosurg (Hagerstown). (2023) 25(3):216–41. 10.1227/ons.0000000000000794 PMC10389757 37387582

[8] Chen H Colasurdo M Malhotra A Gandhi D Bodanapally UK. Advances in chronic subdural hematoma and membrane imaging. Front Neurol. (2024) 15:1366238. 10.3389/fneur.2024.1366238 38725642 PMC11079242

[9] Hamou H Alzaiyani M Pjontek R Kremer B Albanna W Ridwan H. Risk factors of recurrence in chronic subdural hematoma and a proposed extended classification of internal architecture as a predictor of recurrence. Neurosurg Rev. (2022) 45(4):2777–86. 10.1007/s10143-022-01790-8 35461433 PMC9349063

[10] Pahwa B Kazim SF Vellek J Alvarez-Crespo DJ Shah S Tarawneh O. Frailty as a predictor of poor outcomes in patients with chronic subdural hematoma (cSDH): a systematic review of literature. World Neurosurg X. (2024) 23:100372. 10.1016/j.wnsx.2024.100372 38638610 PMC11024655

[11] Von Elm E Altman DG Egger M Pocock SJ Gøtzsche PC Vandenbroucke JP. The strengthening the reporting of observational studies in epidemiology (STROBE) statement: guidelines for reporting observational studies. PLoS Med. (2007) 4(10):e296. 10.1371/journal.pmed.0040296 17941714 PMC2020495

[12] Foppen M Slot KM Vandertop WP Verbaan D. Timing of surgery for chronic subdural hematoma in patients with mild to moderate symptoms: a retrospective cohort study. Acta Neurochir (Wien). (2025) 167(1):147. 10.1007/s00701-025-06552-1 40387935 PMC12089212

[13] Hasan D Nikoubashman O Pjontek R Stockero A Hamou HA Wiesmann M. MRI appearance of chronic subdural hematoma. Front Neurol. (2022) 13:872664. 10.3389/fneur.2022.872664 36003292 PMC9393587

[14] Bechstein M McDonough R Fiehler J Zanolini U Rai H Siddiqui A. Radiological evaluation criteria for chronic subdural hematomas: review of the literature. Clin Neuroradiol. (2022) 32(4):923–9. 10.1007/s00062-022-01138-1 35157088 PMC9744701

[15] Ma R Hao J Ding J Yu H Xia S. Automatic measurement and reference values setting of intracranial cerebrospinal fluid volume: a large-scale analysis of computed tomography images. Quant Imaging Med Surg. (2025) 15(7):6185–99. 10.21037/qims-2024-2571 40727365 PMC12290786

[16] Petrov A Kashevnik A Haleev M Ali A Ivanov A Samochernykh K. AI-based approach to one-click chronic subdural hematoma segmentation using computed tomography images. Sensors (Basel). (2024) 24(3):721. 10.3390/s24030721 38339438 PMC10857356

[17] Han S Feng Y Xu C Li X Zhu F Li Z. Brain re-expansion predict the recurrence of unilateral CSDH: a clinical grading system. Front Neurol. (2022) 13:908151. 10.3389/fneur.2022.908151 36247766 PMC9554254

[18] Koo TK Li MY. A guideline of selecting and reporting intraclass correlation coefficients for reliability research. J Chiropr Med. (2016) 15(2):155–63. 10.1016/j.jcm.2016.02.012 27330520 PMC4913118

[19] Bartek J Biondi A Bonhomme V Castellan L Catapano G Cenzato M. Multidisciplinary consensus-based statement on the current role of middle meningeal artery embolization (MMAE) in chronic SubDural hematoma (cSDH). Brain Spine. (2024) 4:104143. 10.1016/j.bas.2024.104143 39717364 PMC11664065

[20] Mansouri A Nassiri F Scales D Pirouzmand F. Anticoagulation therapy timing in patients with atrial fibrillation after acute and chronic subdural haematoma (ATTAACH): a pilot randomised controlled trial. BMJ Open. (2024) 14(10):e090224. 10.1136/bmjopen-2024-090224 39438108 PMC11499767

[21] Atefi N Alcock S Silvaggio JA Shankar J. Clinical outcome and recurrence risk of chronic subdural hematoma after surgical drainage. Cureus. (2023) 15(2):e35525. 10.7759/cureus.35525 37007398 PMC10054842

[22] Familiari P Lapolla P Relucenti M Battaglione E Cristiano L Sorrentino V. Cortical atrophy in chronic subdural hematoma from ultra-structures to physical properties. Sci Rep. (2023) 13(1):3400. 10.1038/s41598-023-30135-8 36854960 PMC9975247

[23] Liu H Yan R Xie F Richard SA. Hematoma cavity separation and neomembrane thickness are potential triggers of recurrence of chronic subdural hematoma. BMC Surg. (2022) 22(1):236. 10.1186/s12893-022-01687-9 35725399 PMC9210812

[24] Aljabali A Sharkawy AM Jaradat B Serag I Al-dardery NM Abdelhady M. Drainage versus no drainage after burr-hole evacuation of chronic subdural hematoma: a systematic review and meta-analysis of 1,961 patients. Neurosurg Rev. (2023) 46(1):251. 10.1007/s10143-023-02153-7 37726502 PMC10509130

[25] Anagnostopoulos V Brotis AG Tzerefos C Charalambidou A Tasiou A Karavelis A. Valve-controlled chronic subdural hematoma drainage: a feasibility study. Brain Spine. (2022) 2:101693. 10.1016/j.bas.2022.101693 36506285 PMC9729815

[26] Akar O Sucu HK Bozdag S. Simultaneous and consecutive drainage of bilateral chronic subdural hematoma: a randomized controlled trial. Neurol Med Chir (Tokyo). (2024) 64(12):419–27. 10.2176/jns-nmc.2024-0084 39443124 PMC11729258

[27] Tommiska P Knuutinen O Lönnrot K Kivisaari R Raj R Ahmed A. Association between postoperative thromboembolic and hemorrhagic complications and clinical outcomes after surgery for chronic subdural hematoma in patients with anticoagulation therapy for atrial fibrillation. Acta Neurochir (Wien). (2025) 167(1):17. 10.1007/s00701-024-06417-z 39815109 PMC11735564

[28] Vaibhav K Sahu A Prasad RS Deb D Kumar D Karimi A. Prediction of postoperative recurrence of chronic subdural hematoma using preoperative systemic immune inflammation index and eosinophils. Asian J Neurosurg. (2024) 19(4):707–14. 10.1055/s-0044-1789247 39606312 PMC11588619

[29] Hjortdal Grønhøj M Jensen TSR Johannsson B Fugleholm K Rom-Poulsen F. Early spontaneous cessation of subdural drainage after burr hole evacuation of chronic subdural hematoma and risk of recurrence. PLoS One. (2023) 18(5):e0285750. 10.1371/journal.pone.0285750 PMC10191306 37195980

[30] Schack A Olsen MH Hansen JT Magnussen AS Larsen IM Jensen HR. Intracranial pressure changes during early postoperative mobilization in patients with chronic subdural hematoma. Acta Neurochir (Wien). (2025) 167(1):234. 10.1007/s00701-025-06655-9 40888964 PMC12402039

[31] Serag I Abdelhady M Awad AA Wageeh A Shaboub A Elhalag RH. Postoperative elevated bed header position versus supine in the management of chronic subdural hematoma: a systematic review and meta-analysis. Acta Neurol Belg. (2024) 124(4):1177–87. 10.1007/s13760-024-02571-4 38802719 PMC11266250

[32] Blaauw J Hertog HM Holl DC Thüss NS Van Der Gaag NA Jellema K. The cognitive status of chronic subdural hematoma patients after treatment: an exploratory study. Acta Neurochir (Wien). (2023) 165(3):701–9. 10.1007/s00701-023-05508-7 36752891 PMC10006248

[33] Adetunmbi B Adebayo BO Bankole OB Adeyomoye AA Morgan E Kanu OO. Chronic subdural hematoma: predicting postoperative recurrence using a correlation of computerized tomographic volume with intraoperative volume. J West Afr Coll Surg. (2023) 13(4):40–5. 10.4103/jwas.jwas_37_23 38449559 PMC10914102

[34] Imai N Kato T Ito Y Morishima R Aki T Shirakami SI. Timing of chronic subdural hematoma treatment affects middle meningeal artery embolization outcome. Surg Neurol Int. (2024) 15:214. 10.25259/SNI_293_2024 38974558 PMC11225511

[35] Austin PC Lee DS Fine JP. Introduction to the analysis of survival data in the presence of competing risks. Circulation. (2016) 133(6):601–9. 10.1161/CIRCULATIONAHA.115.017719 26858290 PMC4741409

[36] Collins GS Moons KGM Dhiman P Riley RD Beam AL Van Calster B. TRIPOD + AI statement: updated guidance for reporting clinical prediction models that use regression or machine learning methods. Br Med J. (2024) 385:e078378. 10.1136/bmj-2023-078378 PMC11019967 38626948

