# Supplementary material for: Brain re-expansion rate on the first postoperative day and risk of symptomatic recurrence after burr-hole drainage for chronic subdural hematoma
Source: Front Surg. 2026 Oct 21;13:1949842. doi: 10.3389/fsurg.2026.1949842 (PMC13731228; doi:10.3389/fsurg.2026.1949842)
Supplement: Supplementary file 1 [file Table1.docx]

# **Supplementary material**

### **Supplementary Table 1. Strengthening the Reporting of Observational Studies in Epidemiology (STROBE) reporting checklist**

| **Item** | **Domain** | **Reporting requirement** | **Location** |
| --- | --- | --- | --- |
| 1(a) | Title and abstract | Indicate the study design in the title or abstract. | Abstract; 2.1 Study design and participants |
| 1(b) | Title and abstract | Provide an objective, balanced summary of the methods and main findings. | Abstract |
| 2 | Background and rationale | Explain the scientific background and rationale for the study. | 1 Introduction |
| 3 | Objectives | State the objectives and prespecified hypotheses. | Abstract; 1 Introduction |
| 4 | Study design | Present key elements of the study design early in the text. | 2.1 Study design and participants; 2.4 Twenty-four-hour postoperative brain re-expansion rate; 2.5 Outcome definition and landmark follow-up |
| 5 | Setting and dates | Describe the setting, locations, and periods of recruitment, exposure, follow-up, and data collection. | 2.1 Study design and participants |
| 6(a) | Participants | Describe eligibility criteria, patient sources, selection procedures, and follow-up methods. | 2.1 Study design and participants; 2.5 Outcome definition and landmark follow-up; 3.1 Participants and data completeness |
| 6(b) | Matching | For matched designs, describe matching criteria and the number of patients in each group. | Not applicable |
| 7 | Variables | Define outcomes, exposures, predictors, confounders, and diagnostic criteria. | 2.1 Study design and participants; 2.3 Clinical and imaging data; 2.4 Twenty-four-hour postoperative brain re-expansion rate; 2.5 Outcome definition and landmark follow-up; 2.6 Statistical analysis |
| 8 | Data sources and measurement | Describe data sources, measurement methods, and comparability of measurements between groups. | 2.2 Surgery and perioperative management; 2.3 Clinical and imaging data; 2.4 Twenty-four-hour postoperative brain re-expansion rate; 2.5 Outcome definition and landmark follow-up; 3.4 Early postoperative brain re-expansion measures and measurement agreement |
| 9 | Bias | Describe methods used to reduce potential bias. | 2.1 Study design and participants; 2.3 Clinical and imaging data; 2.4 Twenty-four-hour postoperative brain re-expansion rate; 2.5 Outcome definition and landmark follow-up; 2.6 Statistical analysis; 4 Discussion |
| 10 | Study size | Explain how the sample size was determined. | 2.1 Study design and participants; 3.1 Participants and data completeness |
| 11 | Quantitative variables | Explain how continuous variables were handled, including grouping cutoffs and the rationale for grouping. | 2.4 Twenty-four-hour postoperative brain re-expansion rate; 2.6 Statistical analysis; 3.6 Cumulative recurrence by quartiles of brain re-expansion rate; 3.7 Cox regression and restricted cubic spline analyses |
| 12(a) | Statistical methods | Describe statistical methods and methods used to control confounding. | 2.6 Statistical analysis; 3.7 Cox regression and restricted cubic spline analyses |
| 12(b) | Subgroups and interactions | Describe methods for subgroup and interaction analyses. | Not applicable; 3.8 Sensitivity analyses |
| 12(c) | Missing data | Explain how missing data were handled. | 2.1 Study design and participants; 2.6 Statistical analysis; 3.1 Participants and data completeness |
| 12(d) | Incomplete follow-up | Explain how patients lost to follow-up or with unverifiable outcomes were handled. | 2.1 Study design and participants; 2.5 Outcome definition and landmark follow-up; 3.1 Participants and data completeness |
| 12(e) | Sensitivity analyses | Describe methods for sensitivity analyses. | 2.6 Statistical analysis; 3.8 Sensitivity analyses |
| 13(a) | Participant flow | Report the numbers of patients screened, included, undergoing outcome ascertainment, and analyzed. | 3.1 Participants and data completeness |
| 13(b) | Exclusions | Report reasons for exclusion at each stage. | 2.1 Study design and participants; 3.1 Participants and data completeness |
| 13(c) | Flow diagram | Provide a participant flow diagram where appropriate. | 3.1 Participants and data completeness; Figure 1 |
| 14(a) | Descriptive data | Report participant characteristics, exposures, and potential confounders. | 3.2 Clinical characteristics; 3.3 Preoperative imaging and perioperative characteristics; 3.4 Early postoperative brain re-expansion measures and measurement agreement; 3.6 Cumulative recurrence by quartiles of brain re-expansion rate |
| 14(b) | Missing observations | Report the number of missing observations for each variable. | 3.1 Participants and data completeness |
| 14(c) | Follow-up duration | Summarize actual follow-up duration, for example, the mean duration and total person-time. | 2.5 Outcome definition and landmark follow-up; 3.5 Ninety-day postoperative outcomes and reinterventions |
| 15 | Outcome data | Report event counts and outcome measures over time. | 3.5 Ninety-day postoperative outcomes and reinterventions; 3.6 Cumulative recurrence by quartiles of brain re-expansion rate |
| 16(a) | Main estimates | Report unadjusted and adjusted effect estimates and their precision; specify adjustment variables and the rationale for their selection. | 2.6 Statistical analysis; 3.7 Cox regression and restricted cubic spline analyses; 3.8 Sensitivity analyses |
| 16(b) | Category boundaries | Report cutoffs used to categorize continuous variables. | 2.4 Twenty-four-hour postoperative brain re-expansion rate; 3.6 Cumulative recurrence by quartiles of brain re-expansion rate |
| 16(c) | Absolute risk | Where appropriate, consider translating relative risks into absolute risks over a clinically meaningful period. | 3.6 Cumulative recurrence by quartiles of brain re-expansion rate; 3.9 Model discrimination and calibration |
| 17 | Other analyses | Report sensitivity, subgroup, and other supplementary analyses. | 3.3 Preoperative imaging and perioperative characteristics; 3.7 Cox regression and restricted cubic spline analyses; 3.8 Sensitivity analyses; 3.9 Model discrimination and calibration |
| 18 | Key findings | Summarize the main findings in relation to the study objectives. | 4 Discussion; 5 Conclusions |
| 19 | Limitations | Discuss bias, imprecision, and their possible direction and magnitude. | 4 Discussion |
| 20 | Interpretation | Interpret the findings cautiously in light of the objectives, limitations, multiple analyses, and previous evidence. | 4 Discussion; 5 Conclusions |
| 21 | Generalizability | Discuss applicability and external validity. | 4 Discussion; 5 Conclusions |
| 22 | Funding | Disclose funding sources, the role of funders, and, where applicable, funding for the original study. | Funding |

Note: STROBE, Strengthening the Reporting of Observational Studies in Epidemiology. No matching design was used, and no prespecified subgroup effect-modification analyses were performed.

### **Supplementary Table 2. Complete clinical characteristics**

| **Variable** | **Overall** | **No recorded symptomatic recurrence (*n* = 239)** | **Symptomatic recurrence (*n* = 47)** | **Test statistic** | **Raw *P*** | ***P*_FDR_** |
| --- | --- | --- | --- | --- | --- | --- |
| Age, years | 74.4 ± 9.2 | 74.0 ± 9.3 | 76.7 ± 8.1 | *t*(72.0) = −2.036 | 0.045 | 0.195 |
| Male sex, *n* (%) | 196 (68.5) | 163 (68.2) | 33 (70.2) | *χ*^2^(1) = 0.074 | 0.786 | 0.846 |
| Body mass index, kg/m² | 23.9 ± 3.2 (*n* = 281) | 23.8 ± 3.2 (*n* = 235) | 24.2 ± 3.1 (*n* = 46) | *t*(65.3) = −0.850 | 0.398 | 0.600 |
| Smoking history, *n* (%) | 76 (26.6) | 64 (26.8) | 12 (25.5) | *χ*^2^(1) = 0.031 | 0.860 | 0.872 |
| Alcohol consumption, *n* (%) | 54 (18.9) | 46 (19.2) | 8 (17.0) | *χ*^2^(1) = 0.127 | 0.722 | 0.825 |
| Recent head trauma, *n*/*N* (%) | 163/283 (57.6) | 137/237 (57.8) | 26/46 (56.5) | *χ*^2^(1) = 0.026 | 0.872 | 0.872 |
| Hypertension, *n* (%) | 177 (61.9) | 145 (60.7) | 32 (68.1) | *χ*^2^(1) = 0.916 | 0.339 | 0.600 |
| Diabetes mellitus, *n* (%) | 60 (21.0) | 48 (20.1) | 12 (25.5) | *χ*^2^(1) = 0.703 | 0.402 | 0.600 |
| Coronary artery disease, *n* (%) | 55 (19.2) | 44 (18.4) | 11 (23.4) | *χ*^2^(1) = 0.631 | 0.427 | 0.600 |
| Previous stroke, *n* (%) | 49 (17.1) | 39 (16.3) | 10 (21.3) | *χ*^2^(1) = 0.680 | 0.410 | 0.600 |
| Preoperative antithrombotic use, *n* (%) | 79 (27.6) | 61 (25.5) | 18 (38.3) | *χ*^2^(1) = 3.206 | 0.073 | 0.232 |
| Markwalder grade | 1 [1, 2] | 1 [1, 2] | 1 [1, 2] | *U* = 5736.5 | 0.801 | 0.846 |
| Glasgow Coma Scale score | 15 [14, 15] | 15 [14, 15] | 15 [15, 15] | *U* = 5211.5 | 0.332 | 0.600 |
| Platelet count, ×10⁹/L | 212.4 ± 57.9 (*n* = 282) | 215.0 ± 56.6 (*n* = 236) | 199.1 ± 63.3 (*n* = 46) | *t*(59.8) = 1.580 | 0.119 | 0.302 |
| International normalized ratio | 1.05 [0.97, 1.11] (*n* = 283) | 1.05 [0.98, 1.11] (*n* = 236) | 1.02 [0.96, 1.12] (*n* = 47) | *U* = 5799.0 | 0.622 | 0.788 |
| Activated partial thromboplastin time, s | 31.1 ± 4.8 (*n* = 282) | 30.9 ± 4.8 (*n* = 236) | 32.0 ± 4.9 (*n* = 46) | *t*(62.6) = −1.360 | 0.179 | 0.377 |

Note: Data are presented as mean ± standard deviation, median [25th percentile, 75th percentile], or count (percentage). The group without recorded symptomatic recurrence included eight patients who died before recurrence and 231 who remained alive without recurrence. Available sample sizes for body mass index, recent head trauma, platelet count, international normalized ratio, and activated partial thromboplastin time were 281, 283, 282, 283, and 282, respectively; corresponding proportions were calculated using available cases. The symbols *t*, *U*, and *χ*^2^ denote the Welch independent-samples *t* statistic, Mann–Whitney *U* statistic, and Pearson chi-square statistic, respectively; values in parentheses after statistics indicate degrees of freedom. *P* denotes the corresponding test *P* value. *P*_FDR_ denotes the Benjamini–Hochberg-adjusted value across the 38 exploratory comparisons in this table and Supplementary Table 3; FDR, false discovery rate. *n*, number with the characteristic or the indicated analysis sample size; *N*, available sample size; —, not applicable.

### **Supplementary Table 3. Complete imaging and perioperative characteristics**

| **Variable** | **Overall** | **No recorded symptomatic recurrence (*n* = 239)** | **Symptomatic recurrence (*n* = 47)** | **Test statistic** | **Raw *P*** | ***P*_FDR_** |
| --- | --- | --- | --- | --- | --- | --- |
| Hematoma laterality, *n* (%) | — | — | — | *χ*^2^(2) = 4.225 | 0.121 | 0.302 |
| Left | 100 (35.0) | 88 (36.8) | 12 (25.5) | — | — | — |
| Right | 104 (36.4) | 88 (36.8) | 16 (34.0) | — | — | — |
| Bilateral | 82 (28.7) | 63 (26.4) | 19 (40.4) | — | — | — |
| Preoperative hematoma volume on the operated side, mL | 114.2 ± 33.0 | 112.4 ± 32.3 | 123.6 ± 34.8 | *t*(62.6) = −2.034 | 0.046 | 0.195 |
| Maximum hematoma thickness, mm | 18.9 ± 4.4 | 18.8 ± 4.4 | 19.5 ± 4.7 | *t*(62.9) = −0.943 | 0.349 | 0.600 |
| Midline shift, mm | 5.0 [2.9, 6.8] | 4.9 [2.8, 6.6] | 5.4 [3.5, 7.6] | *U* = 4774.0 | 0.104 | 0.302 |
| CT density, *n* (%) | — | — | — | *χ*^2^(3) = 5.699 | 0.127 | 0.302 |
| Hypodense | 52 (18.2) | 46 (19.2) | 6 (12.8) | — | — | — |
| Isodense | 54 (18.9) | 48 (20.1) | 6 (12.8) | — | — | — |
| Hyperdense | 24 (8.4) | 22 (9.2) | 2 (4.3) | — | — | — |
| Mixed density | 156 (54.5) | 123 (51.5) | 33 (70.2) | — | — | — |
| Internal hematoma architecture, *n* (%) | — | — | — | *χ*^2^(3) = 12.099 | 0.007 | 0.038 |
| Homogeneous | 111 (38.8) | 100 (41.8) | 11 (23.4) | — | — | — |
| Laminar | 62 (21.7) | 52 (21.8) | 10 (21.3) | — | — | — |
| Separated | 68 (23.8) | 48 (20.1) | 20 (42.6) | — | — | — |
| Trabecular | 45 (15.7) | 39 (16.3) | 6 (12.8) | — | — | — |
| Moderate-to-severe brain atrophy, *n* (%) | 108 (37.8) | 81 (33.9) | 27 (57.4) | *χ*^2^(1) = 9.273 | 0.002 | 0.018 |
| Anesthetic technique, *n* (%) | — | — | — | *χ*^2^(1) = 0.670 | 0.413 | 0.600 |
| Local anesthesia or sedation | 197 (68.9) | 167 (69.9) | 30 (63.8) | — | — | — |
| General anesthesia | 89 (31.1) | 72 (30.1) | 17 (36.2) | — | — | — |
| Number of burr holes, *n* (%) | — | — | — | *χ*^2^(1) = 0.184 | 0.668 | 0.818 |
| 1 | 214 (74.8) | 180 (75.3) | 34 (72.3) | — | — | — |
| 2 | 72 (25.2) | 59 (24.7) | 13 (27.7) | — | — | — |
| Operated side, *n* (%) | — | — | — | *χ*^2^(2) = 3.769 | 0.152 | 0.340 |
| Left | 112 (39.2) | 98 (41.0) | 14 (29.8) | — | — | — |
| Right | 116 (40.6) | 97 (40.6) | 19 (40.4) | — | — | — |
| Bilateral | 58 (20.3) | 44 (18.4) | 14 (29.8) | — | — | — |
| Drainage duration, h | 33.8 [29.0, 39.3] | 34.1 [29.4, 39.6] | 31.8 [27.4, 37.4] | *U* = 6564.0 | 0.068 | 0.232 |
| Drainage volume during postoperative hours 0–24, mL | 126 [93, 174] | 129 [100, 176] | 94 [73, 153] | *U* = 7112.0 | 0.004 | 0.025 |
| Preoperative antiplatelet interruption, days† | 2.8 [2.4, 3.4] (*n* = 61) | 2.8 [2.4, 3.5] (*n* = 47) | 2.8 [2.5, 2.9] (*n* = 14) | *U* = 350.0 | 0.725 | 0.825 |
| Postoperative interval to antiplatelet resumption, days† | 9.3 [8.0, 10.5] (*n* = 61) | 9.1 [8.0, 10.5] (*n* = 47) | 9.4 [8.5, 10.3] (*n* = 14) | *U* = 349.0 | 0.738 | 0.825 |
| Preoperative anticoagulant interruption, days‡ | 1.9 [1.3, 2.2] (*n* = 18) | 2.0 [1.3, 2.2] (*n* = 14) | 1.5 [1.2, 1.9] (*n* = 4) | *U* = 36.0 | 0.442 | 0.600 |
| Postoperative interval to anticoagulant resumption, days‡ | 8.2 [7.0, 9.7] (*n* = 18) | 8.1 [6.8, 9.5] (*n* = 14) | 9.5 [8.2, 10.5] (*n* = 4) | *U* = 18.0 | 0.327 | 0.600 |
| Interval from surgery completion to postoperative CT, h | 24.0 ± 0.9 | 24.0 ± 0.9 | 23.9 ± 1.0 | *t*(62.9) = 0.650 | 0.518 | 0.679 |
| Total postoperative residual subdural volume, mL | 49.6 [32.2, 71.7] | 46.8 [31.0, 64.8] | 74.5 [49.2, 106.9] | *U* = 3259.0 | <0.001 | <0.001 |
| Subdural air volume, mL | 9.5 [7.2, 13.3] | 9.3 [6.9, 13.2] | 10.0 [8.6, 13.6] | *U* = 4631.0 | 0.057 | 0.218 |
| Postoperative residual fluid volume, mL | 38.4 [21.2, 60.6] | 35.9 [20.5, 55.2] | 62.8 [38.8, 94.6] | *U* = 3434.0 | <0.001 | <0.001 |
| 24-hour brain re-expansion rate, %/24 h | 54.2 [39.7, 67.2] | 56.8 [43.4, 68.8] | 40.8 [26.3, 54.3] | *U* = 7884.0 | <0.001 | <0.001 |
| Brain re-expansion rate excluding air, %/24 h | 65.0 [49.7, 78.1] | 66.8 [52.0, 79.7] | 50.8 [33.4, 69.8] | *U* = 7584.0 | <0.001 | 0.001 |

Note: Continuous variables are presented as mean ± standard deviation or median [25th percentile, 75th percentile], and categorical variables as counts (percentages). The group without recorded symptomatic recurrence included eight patients who died before recurrence and 231 who remained alive without recurrence. †The antiplatelet subgroup comprised 61 patients: 47 without recorded recurrence and 14 with recurrence. ‡The anticoagulant subgroup comprised 18 patients: 14 without recorded recurrence and four with recurrence. There was no overlap between the two medication groups. Medication-timing comparisons used the relevant subgroup denominators; anticoagulant comparisons used the two-sided exact Mann–Whitney *U* test. The symbols *t*, *U*, and *χ*^2^ denote the Welch independent-samples *t* statistic, Mann–Whitney *U* statistic, and Pearson chi-square statistic, respectively; values in parentheses after statistics indicate degrees of freedom. Tests for variables with multiple categories assess the overall distribution. *P* denotes the corresponding test *P* value; *P*_FDR_ denotes the Benjamini–Hochberg-adjusted value across the same 38 comparisons as Supplementary Table 2. CT, computed tomography; FDR, false discovery rate; *n*, number of patients; —, not applicable.

### **Supplementary Table 4. Postoperative mobilization timing, drainage dynamics, and symptomatic recurrence**

| **Measure** | **Mobilization within 24 h** | **Delayed mobilization** | **Effect estimate (95% CI) or test statistic** | ***P*** | **Statistical method** |
| --- | --- | --- | --- | --- | --- |
| Sample size | 232 | 54 | — | — | — |
| Drainage volume during postoperative hours 0–24, mL | 125 [93, 173] | 137 [93, 177] | *U* = 6075.0 | 0.731 | Mann–Whitney *U* test |
| 24-hour brain re-expansion rate, %/24 h | 55.7 [40.2, 67.3] | 50.7 [37.5, 62.4] | *U* = 6808.0 | 0.321 | Mann–Whitney *U* test |
| Mobilization timing and brain re-expansion rate | — | — | Adjusted difference: 5.1 percentage points/24 h (−2.2 to 12.3) | 0.172 | Median regression |
| Mobilization timing and drainage volume | — | — | Geometric mean ratio: 0.97 (0.85–1.11) | 0.637 | Log-linear regression |
| Brain re-expansion rate, per 10-percentage-point/24-h increase | — | — | Adjusted HR: 0.74 (0.64–0.85) | <0.001 | Jointly stratified cause-specific Cox model |
| Mobilization within 24 h | — | — | Adjusted HR: 1.31 (0.62–2.78) | 0.485 | Jointly stratified cause-specific Cox model |

Note: Data are presented as median [25th percentile, 75th percentile]; between-group comparisons use the two-sided Mann–Whitney *U* test. The median-regression difference is early minus delayed mobilization and uses asymptotic robust standard errors. The geometric mean ratio from log-linear regression compares early with delayed mobilization and uses the heteroskedasticity-consistent covariance estimator type 3 (HC3). Both models adjust for age, admission Markwalder grade, anesthetic technique, bilateral disease, moderate-to-severe brain atrophy, and preoperative volume. The mobilization-related cause-specific Cox model is jointly stratified by brain atrophy and antithrombotic use and includes brain re-expansion rate, age, bilateral disease, preoperative volume, and mobilization timing. Risk-set entry occurs at the later of the first postoperative computed tomography (CT) or 24 h after surgery. The eight deaths before recurrence are censored on the date of death. HR, cause-specific hazard ratio; CI, confidence interval; *P*, two-sided *P* value; *U*, Mann–Whitney *U* statistic; —, not applicable.

### **Supplementary Table 5. Tests of proportional hazards and proportional subdistribution hazards**

| **Model** | **Variable** | **Cox *χ*^2^** | **Cox *P*** | **Fine–Gray *χ*^2^** | **Fine–Gray *P*** |
| --- | --- | --- | --- | --- | --- |
| Unstratified model | Brain re-expansion rate | 1.941 | 0.164 | 1.999 | 0.157 |
| Unstratified model | Age | 0.063 | 0.802 | 0.062 | 0.803 |
| Unstratified model | Preoperative antithrombotic use | 3.315 | 0.069 | 3.385 | 0.066 |
| Unstratified model | Bilateral disease | 0.056 | 0.812 | 0.059 | 0.808 |
| Unstratified model | Moderate-to-severe brain atrophy | 6.612 | 0.010 | 6.664 | 0.010 |
| Unstratified model | Preoperative hematoma volume | 1.015 | 0.314 | 1.015 | 0.314 |
| Unstratified model | Global (6 degrees of freedom) | 14.310 | 0.026 | 14.521 | 0.024 |
| Stratified by brain atrophy alone | Brain re-expansion rate | 0.974 | 0.324 | 1.004 | 0.316 |
| Stratified by brain atrophy alone | Age | 0.040 | 0.842 | 0.043 | 0.836 |
| Stratified by brain atrophy alone | Preoperative antithrombotic use | 4.634 | 0.031 | 4.738 | 0.030 |
| Stratified by brain atrophy alone | Bilateral disease | 0.000 | 0.996 | 0.000 | 0.997 |
| Stratified by brain atrophy alone | Preoperative hematoma volume | 0.734 | 0.391 | 0.737 | 0.391 |
| Stratified by brain atrophy alone | Global (5 degrees of freedom) | 7.072 | 0.215 | 7.226 | 0.204 |
| Jointly stratified model | Brain re-expansion rate | 1.247 | 0.264 | 1.280 | 0.258 |
| Jointly stratified model | Age | 0.006 | 0.938 | 0.006 | 0.940 |
| Jointly stratified model | Bilateral disease | 0.203 | 0.653 | 0.212 | 0.645 |
| Jointly stratified model | Preoperative hematoma volume | 1.543 | 0.214 | 1.555 | 0.212 |
| Jointly stratified model | Global (4 degrees of freedom) | 2.844 | 0.584 | 2.902 | 0.574 |

Note: Variable-specific tests have 1 degree of freedom; global tests for the three models have 6, 5, and 4 degrees of freedom, respectively. *χ*^2^ denotes the efficient score test statistic for the interaction between a covariate and centered log(postoperative days), and *P* denotes the corresponding chi-square test *P* value; the other coefficients are accounted for as nuisance parameters. Cox tests use the cause-specific risk set; Fine–Gray tests use the extended subdistribution risk set including competing deaths. Common-coefficient tests are not performed for stratification variables.

### **Supplementary Table 6. Sensitivity analyses**

| **Analysis and effect unit** | ***n*** | **Recurrence** | **Death** | **Effect estimate (95% CI)** | ***z*** | ***P*** |
| --- | --- | --- | --- | --- | --- | --- |
| Primary jointly stratified cause-specific Cox model; per 10-percentage-point/24-h increase | 286 | 47 | 8 | HR = 0.74 (0.64–0.85) | −4.147 | <0.001 |
| Jointly stratified Fine–Gray model; per 10-percentage-point/24-h increase | 286 | 47 | 8 | sHR = 0.74 (0.65–0.85) | −4.296 | <0.001 |
| Unstratified cause-specific Cox model; per 10-percentage-point/24-h increase | 286 | 47 | 8 | HR = 0.74 (0.64–0.85) | −4.180 | <0.001 |
| Residual fluid only; per 10-percentage-point/24-h increase | 286 | 47 | 8 | HR = 0.77 (0.67–0.89) | −3.633 | <0.001 |
| Relative volume reduction without time standardization; per 10-percentage-point increase; additionally adjusted for CT interval | 286 | 47 | 8 | HR = 0.74 (0.64–0.85) | −4.182 | <0.001 |
| Total postoperative residual subdural volume; per 10-mL increase | 286 | 47 | 8 | HR = 1.27 (1.14–1.42) | 4.348 | <0.001 |
| Absolute brain re-expansion rate; per 10-mL/24-h increase | 286 | 47 | 8 | HR = 0.79 (0.71–0.88) | −4.289 | <0.001 |
| Excluding bilateral disease treated unilaterally; per 10-percentage-point/24-h increase | 262 | 42 | 7 | HR = 0.74 (0.63–0.86) | −3.910 | <0.001 |
| 90-day binary logistic regression; per 10-percentage-point/24-h increase | 286 | 47 | 8 | OR = 0.70 (0.59–0.83) | −4.012 | <0.001 |

Note: All models control for age, preoperative antithrombotic use, bilateral disease, moderate-to-severe brain atrophy, and preoperative volume. Except for the unstratified model, survival models are jointly stratified by brain atrophy and antithrombotic use; the logistic model represents the four-stratum structure using three indicator variables. The model without time standardization additionally adjusts for the computed tomography (CT) interval. HR, cause-specific hazard ratio; sHR, subdistribution hazard ratio; OR, odds ratio; CI, confidence interval; *z*, Wald statistic; *P*, two-sided Wald test *P* value; *n*, analysis sample size. All deaths occurred before recurrence: Cox models censor at the date of death, Fine–Gray models treat death as a competing event, and the logistic outcome is coded as 0. After exclusion of 24 patients, the remaining 262 comprised 42 with recurrence, seven who died before recurrence, and 213 who remained alive without recurrence.

### **Supplementary Table 7. Jointly stratified Fine–Gray regression analysis of 90-day symptomatic recurrence**

| **Variable** | ***β*** | **Robust standard error** | **Adjusted sHR (95% CI)** | ***z*** | ***P*** |
| --- | --- | --- | --- | --- | --- |
| Brain re-expansion rate, per 10-percentage-point/24-h increase | −0.30081163 | 0.07002530 | 0.74 (0.65–0.85) | −4.296 | <0.001 |
| Age, per 10-year increase | 0.38287809 | 0.15414844 | 1.47 (1.08–1.98) | 2.484 | 0.013 |
| Bilateral disease | 0.75401077 | 0.30709930 | 2.13 (1.16–3.88) | 2.455 | 0.014 |
| Preoperative hematoma volume, per 10-mL increase | 0.08495459 | 0.04897043 | 1.09 (0.99–1.20) | 1.735 | 0.083 |

**Baseline cumulative subdistribution hazards by joint stratum**

| **Moderate-to-severe brain atrophy** | **Preoperative antithrombotic use** | ***n*** | **Recurrence** | **Death** | ***H*_0_*_s_*(90)** |
| --- | --- | --- | --- | --- | --- |
| 0 | 0 | 126 | 12 | 4 | 0.29852759 |
| 0 | 1 | 52 | 8 | 2 | 0.53857265 |
| 1 | 0 | 81 | 17 | 2 | 0.58362526 |
| 1 | 1 | 27 | 10 | 0 | 1.53121662 |

Note: The model includes 286 patients: 47 with recurrence, eight who died before recurrence, and 231 who remained alive without recurrence. It is stratified by the four combinations of moderate-to-severe brain atrophy and preoperative antithrombotic use and adjusted for age, bilateral disease, and preoperative volume. *β* denotes the regression coefficient; robust standard errors are derived from the patient-level sandwich covariance matrix. sHR, subdistribution hazard ratio, equal to exp(*β*); CI, confidence interval; *z*, ratio of *β* to its standard error; *P*, two-sided Wald test *P* value. *H*_0_*_s_*(90) is the 90-day baseline cumulative subdistribution hazard in stratum s. In the stratum table, 0 and 1 indicate absence and presence of the corresponding characteristic; death denotes death before recurrence. The effect unit for brain re-expansion rate is a 10-percentage-point/24-h increase. Age and volume are coded using the centering described in the main text. *n* denotes the number of patients in each stratum.
